# Mapping the Aetiology of Non-Malarial Febrile Illness in Southeast Asia through a Systematic Review—Terra Incognita Impairing Treatment Policies

**DOI:** 10.1371/journal.pone.0044269

**Published:** 2012-09-06

**Authors:** Nathalie Acestor, Richard Cooksey, Paul N. Newton, Didier Ménard, Philippe J. Guerin, Jun Nakagawa, Eva Christophel, Iveth J. González, David Bell

**Affiliations:** 1 Malaria/Acute Febrile Syndrome Programme, Foundation for Innovative New Diagnostics (FIND), Geneva, Switzerland; 2 Centre for Clinical Vaccinology and Tropical Medicine, Nuffield Department of Clinical Medicine, University of Oxford, Churchill Hospital, Oxford, United Kingdom; 3 WorldWide Antimalarial Resistance Network, University of Oxford, Churchill Hospital, Oxford, United Kingdom; 4 Wellcome Trust-Mahosot-Oxford Tropical Medicine Research Collaboration, Microbiology Laboratory, Mahosot Hospital, Vientiane, Lao PDR; 5 Malaria Molecular Epidemiology Unit, Pasteur Institute of Cambodia, Phnom Penh, Cambodia; 6 Malaria, Other Vector-borne and Parasitic Diseases, WHO Regional Office for the Western Pacific, Manila, The Philippines; University of Barcelona, Spain

## Abstract

**Background:**

An increasing use of point of care diagnostic tests that exclude malaria, coupled with a declining malaria burden in many endemic countries, is highlighting the lack of ability of many health systems to manage other causes of febrile disease. A lack of knowledge of distribution of these pathogens, and a lack of screening and point-of-care diagnostics to identify them, prevents effective management of these generally treatable contributors to disease burden. While prospective data collection is vital, an untapped body of knowledge already exists in the published health literature.

**Methods:**

Focusing on the Mekong region of Southeast Asia, published data from 1986 to 2011 was screened to for frequency of isolation of pathogens implicated in aetiology of non-malarial febrile illness. Eligibility criteria included English-language peer-reviewed studies recording major pathogens for which specific management is likely to be warranted. Of 1,252 identified papers, 146 met inclusion criteria and were analyzed and data mapped.

**Results:**

Data tended to be clustered around specific areas where research institutions operate, and where resources to conduct studies are greater. The most frequently reported pathogen was dengue virus (n = 70), followed by *Orientia tsutsugamushi* and *Rickettsia* species (scrub typhus/murine typhus/spotted fever group n = 58), *Leptospira* spp. (n = 35), *Salmonella enterica* serovar Typhi and Paratyphi (enteric fever n = 24), *Burkholderia pseudomallei* (melioidosis n = 14), and Japanese encephalitis virus (n = 18). Wide tracts with very little published data on aetiology of fever are apparent.

**Discussion and Conclusions:**

This mapping demonstrates a very heterogeneous distribution of information on the causes of fever in the Mekong countries. Further directed data collection to address gaps in the evidence-base, and expansion to a global database of pathogen distribution, is readily achievable, and would help define wider priorities for research and development to improve syndromic management of fever, prioritize diagnostic development, and guide empirical therapy.

## Introduction

Millennium development goal 4 (MDG4) aims for reduction in infant and childhood mortality by two thirds, between 1990 and 2015 [1]. Achieving this in most low-income countries will require major reductions in mortality due to infectious diseases. This aim drives the current high resource allocation for malaria interventions in aid budgets, associated in recent years with considerable reduction in reported malaria mortality [2]. As malaria management transitions from symptom-based to parasite-based diagnosis, fuelled by increased resources and the tightening of the World Health Organization (WHO) recommendations on parasite-based diagnosis prior to treatment [3], it is apparent that non-malarial fevers are major, uncounted and neglected causes of morbidity and mortality [4], [5], [6]. By ‘non-malaria febrile illness (NMFI)’, we refer here to infectious diseases in patients who present with undifferentiated fever and require malaria rapid diagnostic tests (RDTs)/microscopy - but in whom these tests are negative. Much of what was diagnosed as malaria in the past, on basis of symptoms and signs, was probably not malaria but caused by a wide variety of pathogens that remained untreated by antimalarial therapies [4], [7], [8]. Hence, a paradox of modern malaria control in the age of artemisinin combination therapies (ACTs) is no longer what antimalarials to use, but how to manage patients presenting with fever not due to malaria.

Recent data suggests that, with the current level of intervention, MDG4 will not be met. Progress is particularly poor in sub-Saharan Africa where recent increases in anti-malaria resources have been concentrated [1], [2]. Addressing the burden of non-malarial fever will be essential to regaining momentum and achieving this global goal. This will require a shift in emphasis from a vertical approach to malaria to a more syndromic approach, managing malaria as one of many treatable causes of fever, and utilizing RDTs as a tool to guide effective management of both malaria-positive and malaria-negative cases.

Although NMFI causes higher mortality than malaria globally [9], including in malaria-endemic countries [4], allocation of resources to both research and development, and implementation, is small. There is no Global Fund equivalent to channel implementation funding to disease programmes as is the case for malaria, TB and HIV, and combined research and development funding for malaria alone equals that of the first 10 ‘neglected’ (non-TB and HIV) infectious diseases [10]. This lack of interest has resulted in a lack of data on probable causes and lack of specific low-cost point of care diagnostics and surveillance tools. These important gaps limit the ability of health services to address non-malarial morbidity and mortality through effective case management.

**Table 1:** Criteria used for selection of papers for the mapping of aetiology of non-malaria febrile illness in Southeast Asia.

Inclusion criteria	Study conducted in the Greater Mekong region (Cambodia, Laos, Vietnam, Thailand, Myanmar and the Yunnan Province of the People’s Republic of China).
	Study including patients (or a clear subset of patients).
	Patients tested by laboratory analysis (serology, molecular methods, culture or antigen tests) performed in a laboratory setting.
Exclusion criteria	No abstract or free full text access available.
	Editorial or opinion rather than publication of new data.
	Economic impact studies of febrile illnesses without pathological testing.
	Follow-up studies (cohort of patients already described in a previous study).
	Animal studies.
	Experimental (*in vitro* or *in vivo* cellular, molecular, biochemical studies or other studies that do not involve patients).
	Vaccine and drug trials.
	Modeling studies.
	Laboratory methods descriptions.
	Case reports and studies of biochemical or clinical outcomes only (e.g. liver function, pathology, disease features, clinical presentation of a disease).
	Biomarker evaluation.
	Travel (acute febrile illnesses associated with a travel episode, as place of infection may be in doubt).
	Other studies of disease not including identification of causes of fever (e.g. demographic surveillance, geographic information, vector transmission, climate impact).

Understanding the major causes of NMFI will enable health services to prioritize management strategies likely to have the greatest impact on patient outcomes when malaria and other diagnosable causes are treated. Understanding the impact of aetiological agents on a larger scale will facilitate prioritization of development of pathogen-specific or multiplexed diagnostics to optimize management. While a number of studies looking specifically at non-malarial fever aetiology are reported or underway, these are high cost projects and the results may be specific only to the geographical area of the study. However, a body of data on the aetiology of fever has been published that will help inform the likely causes of NMFI. Making such data accessible and mapping pathogens and their antimicrobial resistance patterns, could serve to guide targeting of further studies and initial steps to prioritizing patient management.

In much of Southeast Asia, malaria is a relatively minor and reducing contributor to mortality [2], [11], [12], [13], with transmission heterogeneous and often absent at a local scale [14], [15], but remains a major public health priority due to concern over resurgence, and emergence of parasite resistance to artemisinin derivatives that could jeopardize recent progress [16], [17]. In order to sustain funding to maintain pressure against transmission, malaria programmes will need to also effectively address other major causes of treatable illness and so provide immediate public health benefit.

We therefore undertook a review of published studies to record and map the identification of major pathogens causing treatable febrile illness, or commonly implicated in febrile disease outbreaks, in this region. The project aimed to determine the feasibility of establishing an accessible database of febrile disease aetiology based initially on existing published data, and of extending this as a global resource.

**Figure 1:**
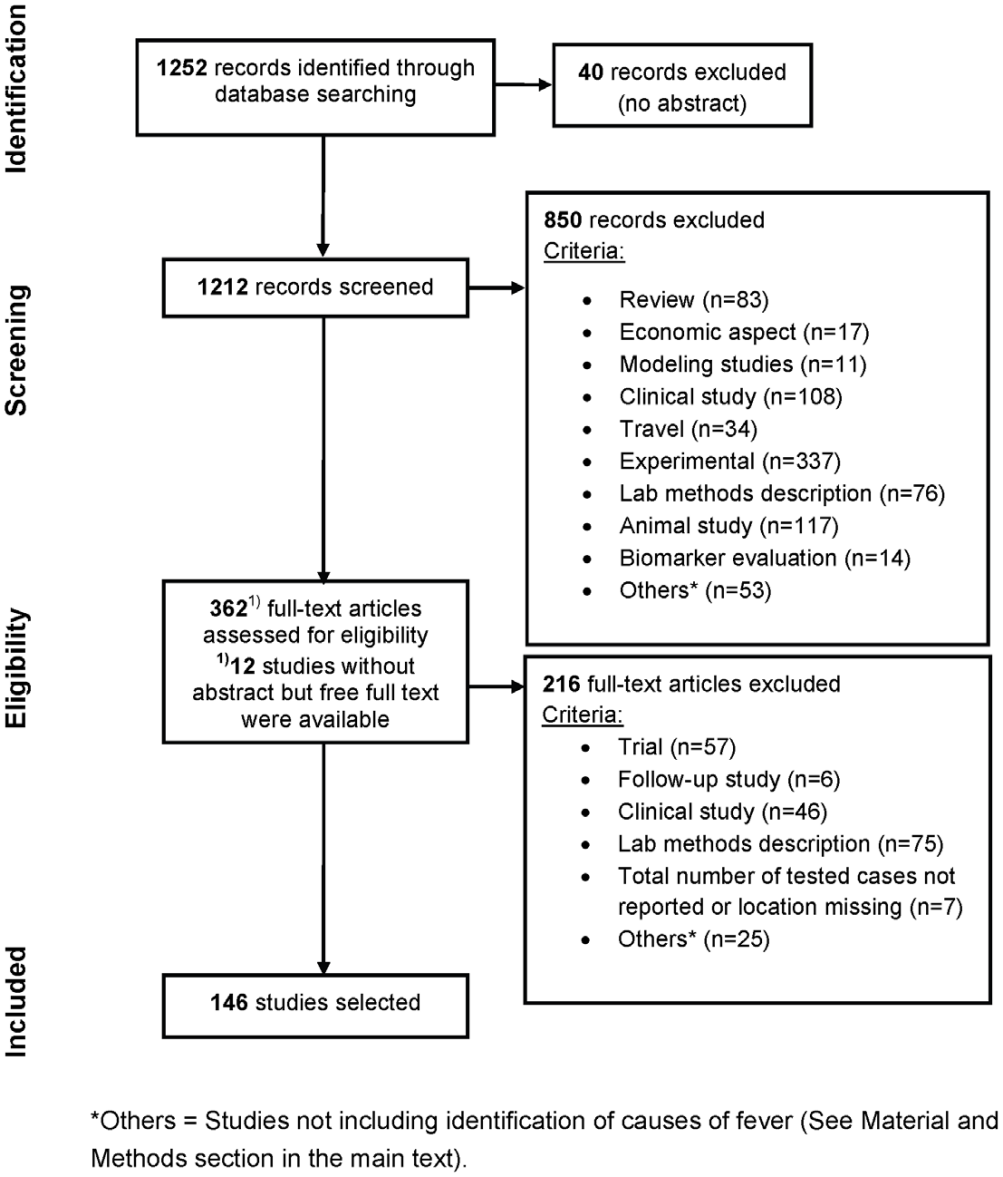
PRISMA flow chart for literature search.

## Methods

### Search Method for Identification of Studies

For this pilot study, the search for data was confined to published studies in countries of the Greater Mekong region; A PubMed search was performed using the keywords “Cambodia” or ‘Lao PDR’ (and ‘Laos’) or ‘Viet Nam’ (and ‘Vietnam’)’ or ‘Myanmar’ (and ‘Burma’) or ‘Thailand’ or ‘Yunnan Province’ (of the People’s Republic Of China), coupled separately with individual search terms for diseases as follows; ‘rickettsial infections’ (search terms: ‘rickett*’, ‘scrub typhus’, ‘murine typhus’, ‘spotted fever group rickett*’), ‘leptospirosis’, ‘typhoid fever’, ‘dengue ’, ‘melioidosis’, and ‘Japanese encephalitis’ (asterisk ‘*’ as truncation symbol). Where studies identified other pathogens in addition to the target pathogens above, these were also included in the database. The search for these further pathogens is therefore less complete. The search was not limited by study design or patient age, but was restricted to articles published in English and within the last twenty-five years (from January 1^st^ 1986 until June 9^th^ 2011). Data were derived from studies using both community-based and hospital-based sampling. Clinical criteria were not included; the review aimed to identify pathogen presence rather than clinical evidence of infection. The proportion of fever aetiology identified in each study was recorded as the main outcome measure. Titles and abstracts were screened for compliance with these inclusion criteria, and then full papers reviewed. Data from unpublished studies were not included.

**Figure 2:**
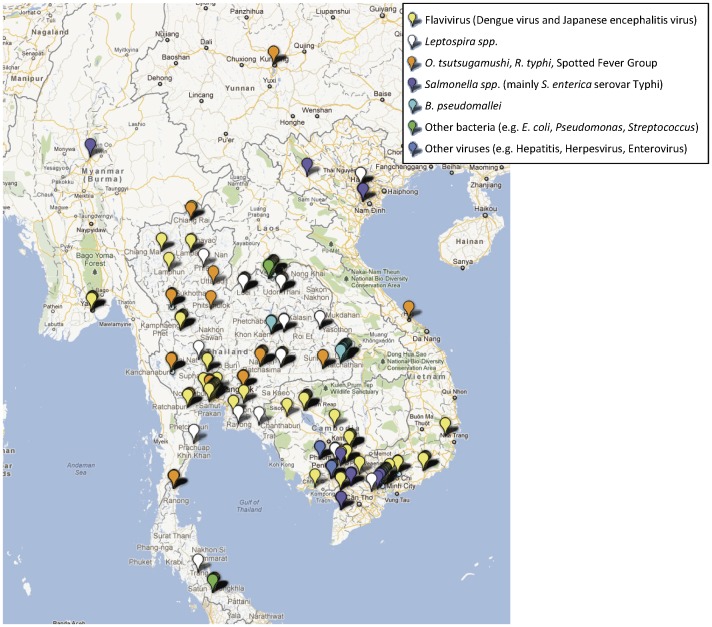
Study sites in the Mekong region where major aetiologies of infectious febrile illnesses have been reported.

### Study Selection and Data Extraction

Criteria for inclusion and exclusion of studies for this review are listed in Table 1. One author (NA) independently applied these criteria to all identified studies. The following twelve variables were then extracted from each included paper: i) pathogen, ii) type of study, iii) start and end year of the study, iv) country, v) province, vi) patient age (minimum and maximum age), vii) type of patient sample, viii) number of tested cases, ix) number of positive cases, x) method of laboratory analysis, xi) name of the first and last author, and xii) year and the Uniform Resource Locator (URL) link of the publication. Frequency of pathogens was recorded as zero if the study specifically tested for them but they were not detected, but no result was recorded if specific testing was not recorded.

Data were further divided on the basis of recording of demonstrated ‘frequency of acute infection aetiology’, and on the basis of evidence of current or very recent infection (demonstration of specific antigen, nucleic acid, IgM or 4 fold increase in IgG) or ‘frequency of past infection with pathogen’ where evidence of current or very recent infection is not proven (demonstration of specific IgG at only one time point for each patient).

**Figure 3:**
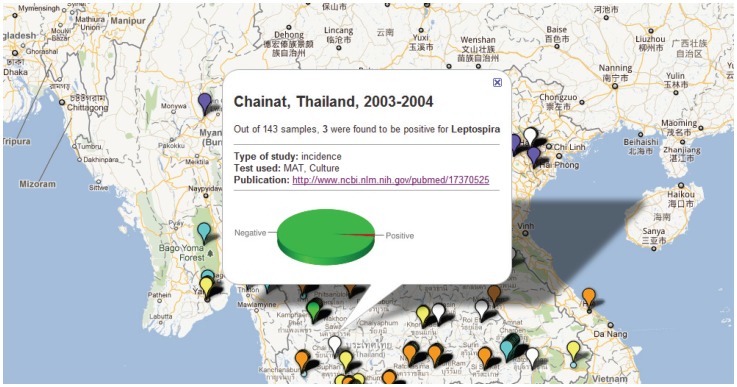
Pop-up window of map showing study sites in the Mekong region where major aetiologies of febrile illnesses have been reported.

### Database

A normalized Microsoft Office (MS) Access 2007 relational database was created with the information obtained from the extracted variables.

### Mapping

To be recorded as a specific point, each study site location was geo-coded to find its latitude and longitude using a web tool (http://stevemorse.org/jcal/latlon.php). Queries were run in MS Access to retrieve data views for import into Google Fusion Tables™ (http://www.google.com/fusiontables/public/tour/index.html). Google Fusion Tables was used to generate individual maps of the distribution of pathogens present in the Mekong region. As study sites with an identical latitude and longitude would be overwritten on a Google Map, a simple algorithm was used to marginally shift (dither) map markers to allow multiple studies from a single site to be displayed - introducing a maximum error of 1.28 km from the geo-located origin.

**Figure 4:**
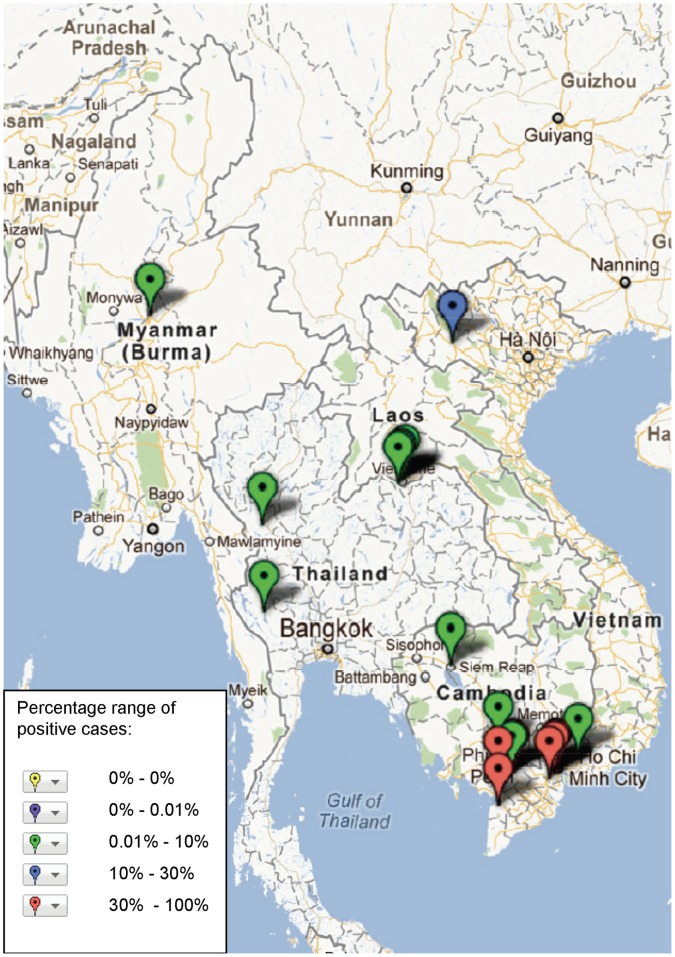
Study sites in the Mekong region where *S.* Typhi has been reported, illustrating frequency of identification (case) based on assays and sample population used in the study (see Table S1).

The final maps include only ‘frequency of acute infection aetiology’ data, excluding papers where only IgG levels without a significant rise in titre are presented, as such cases are probably not related to the aetiology of the presenting febrile episode. These data are coded in categories of frequency with which pathogens *were isolated* by the techniques used in the particular study, on the sample population used. These provide a guide to frequency of isolation, and therefore a rough guide of likelihood of encountering the pathogens, but should not be confused with prevalence or frequency of infection in view of the wide variation in standards and sampling used and broad time-frame. It is noted that some acute secondary dengue infections with a single high IgG titre could be excluded by these criteria.

**Figure 5:**
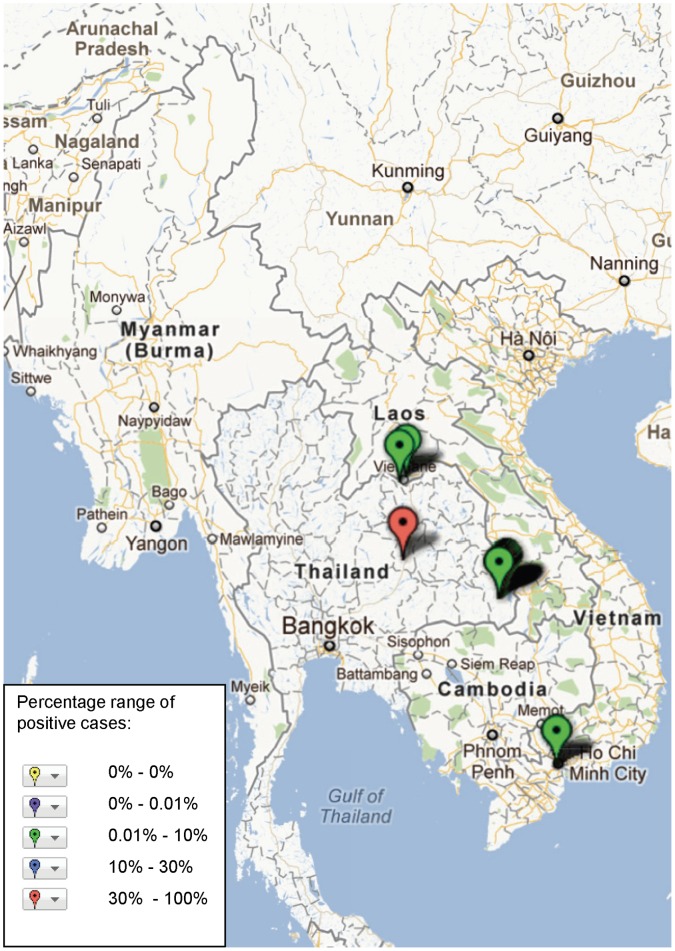
Study sites in the Mekong region where *B. pseudomallei* has been reported, illustrating frequency of identification (case) based on assays and sample population used in the study (see Table S1).

## Results

The initial search of the PubMed database identified 1,252 citations (Figure 1). On initial screening 40 were discarded for having no abstract and 850 studies were discarded because after reviewing the abstracts they did not meet the inclusion criteria. A full-text review led to the exclusion of a further 216 papers, including seven studies with potentially relevant data that were excluded as the total number of patients tested or a precise location for the study was not reported, or artificially-seeded samples (induced infections) were included [18], [19], [20], [21], [22], [23], [24].

**Figure 6:**
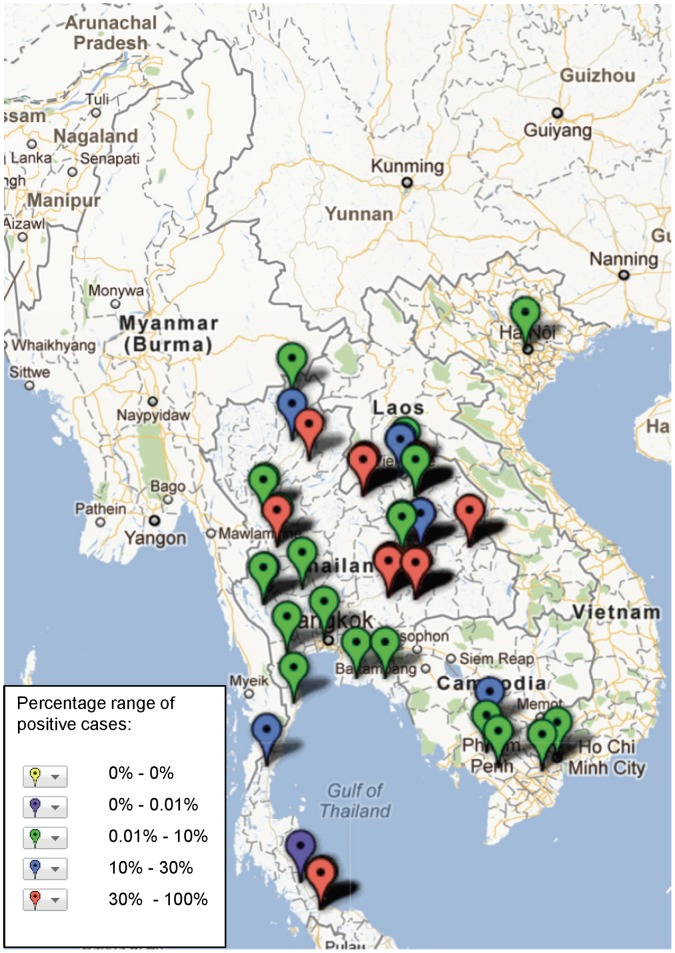
Study sites in the Mekong region where *Leptospira* spp. have been reported, illustrating frequency of identification (case) based on assays and sample population used in the study (see Table S1).

Of 146 studies remaining in the analysis (three covering sites in more than one country), 37 were conducted up to the year 2000 and 109 from 2001 onwards. The largest proportion of these presented data from Thailand (n = 76), then Viet Nam (n = 40), Laos (n = 16) and Cambodia (n = 12) whereas studies with data from Myanmar and the Yunnan Province were particularly lacking (n = 4 and n = 1, respectively). Ninety three studies included patients of all ages (range: 0–100 years), while 42 studies included children only (age <5 years, n = 4; age <10 years, n = 2; age <18 years, n = 36). Three studies included only patients from age 0 to age 20 while eight other studies included only patients >18 years old. The most frequently reported pathogen was dengue virus (n = 70), followed by typhus (scrub typhus/murine typhus/SFG n = 58), *Leptospira* spp. (n = 35), *S*. *typhi* and *S. paratyphi* (enteric fever n = 24), *Burkholderia pseudomallei* (melioidosis n = 14), and Japanese encephalitis virus (n = 18) (Table S1). PubMed-Medline references for these papers are provided in Table S2.

**Figure 7:**
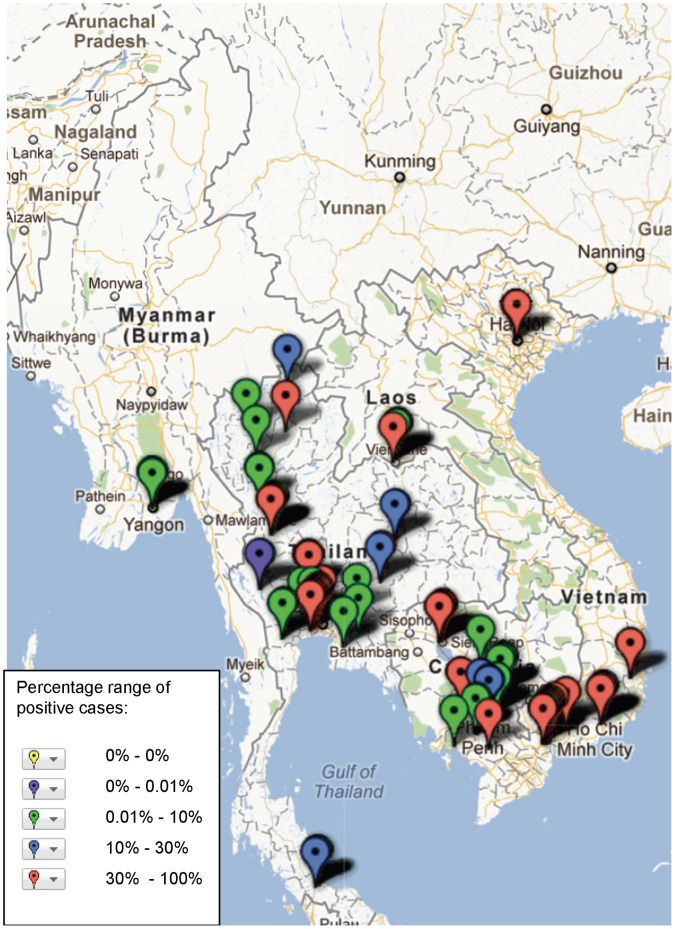
Study sites in the Mekong region where Dengue virus has been reported, illustrating frequency of identification (case) based on assays and sample population used in the study (see Table S1).

Of the 146 studies, 124 included evidence of current or very recent infection (Table S1 **)** and are mapped in Figure 2. Twenty-three studies reported data on frequency of past infection with pathogen (specific IgG at one time point per patient or without significant rise in titre), and these data were excluded from subsequent mapping (Table S3). Each study is mapped with basic descriptive data including study type and frequency of a positive result that can be obtained by clicking the marker on the interactive map (see http://www.finddiagnostics.org/programs/malaria-afs/nmfi-mapping and http://www.wwarn.org/about-us/beyond-malaria ), illustrated by the example in Figure 3.

**Figure 8:**
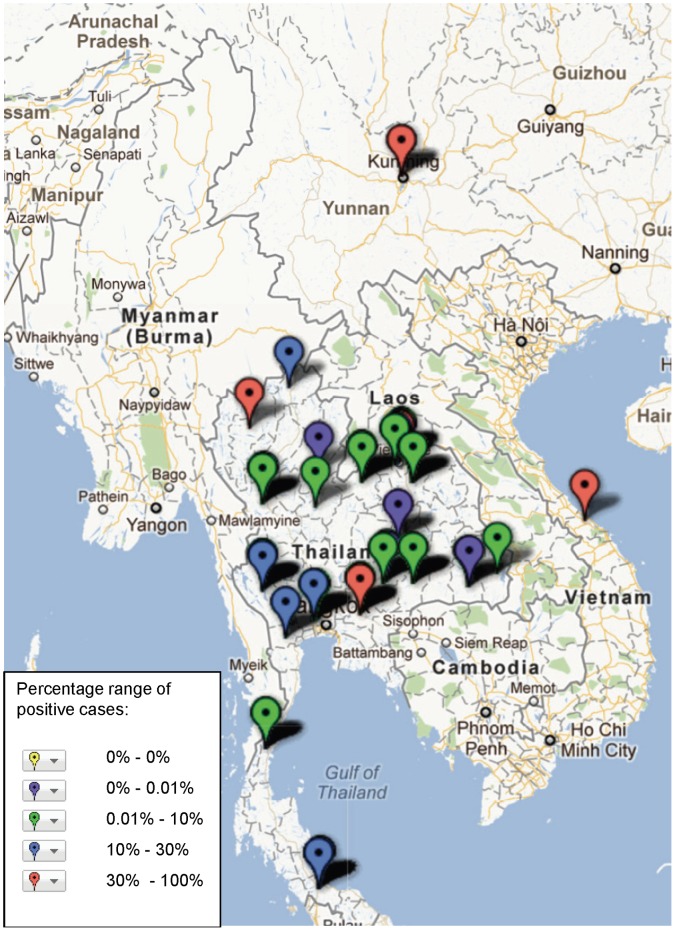
Study sites in the Mekong region where Rickettsioses have been reported, illustrating frequency of identification (case) based on assays and sample population used in the study (see Table S1).

**Figure 9:**
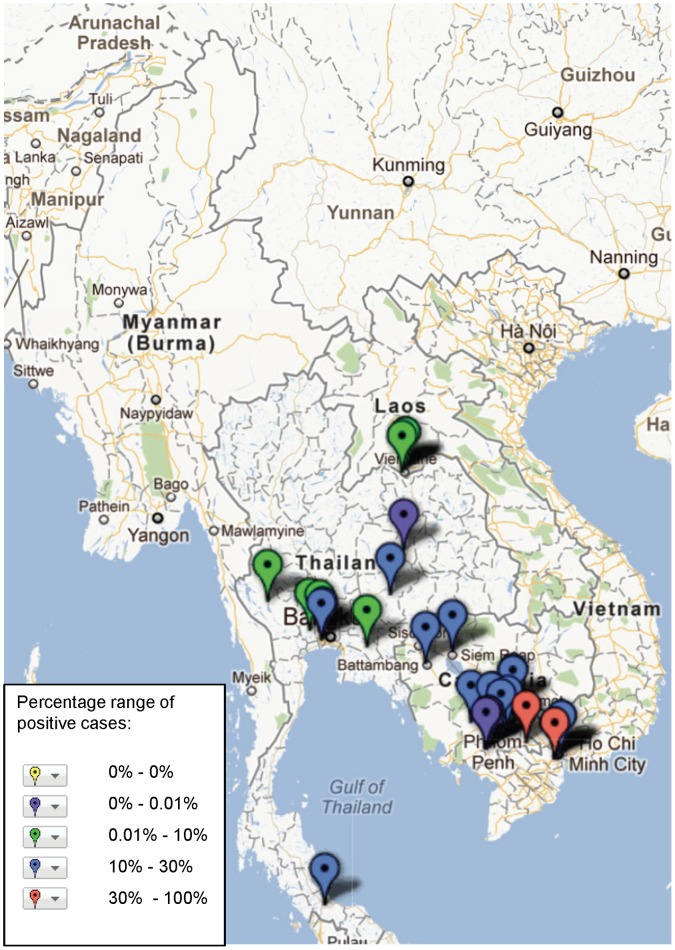
Study sites in the Mekong region where Japanese encephalitis virus has been reported, illustrating frequency of identification (case) based on assays and sample population used in the study (see Table S1).

**Figure 10:**
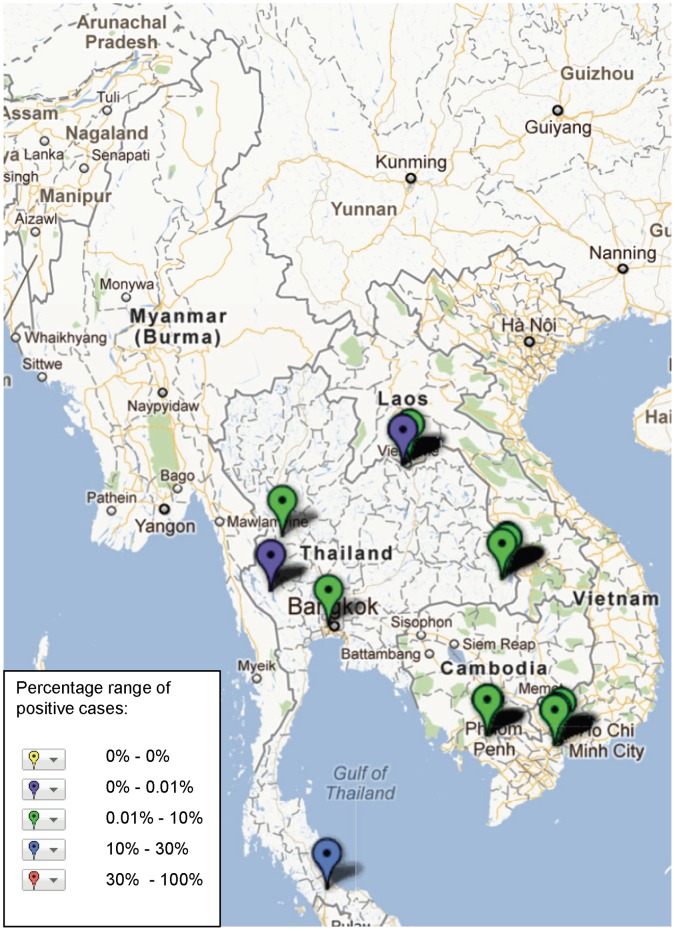
Study sites in the Mekong region where other bacteria (e.g. *E. coli* and *Pseudomonas*, *Streptococcus*, *Staphylococcus*, *Shigella*, *Klebsiella*, *Haemophilus* species) have been reported, illustrating frequency of identification (case) based on assays and sample population used in the study (see Table S1).

**Figure 11:**
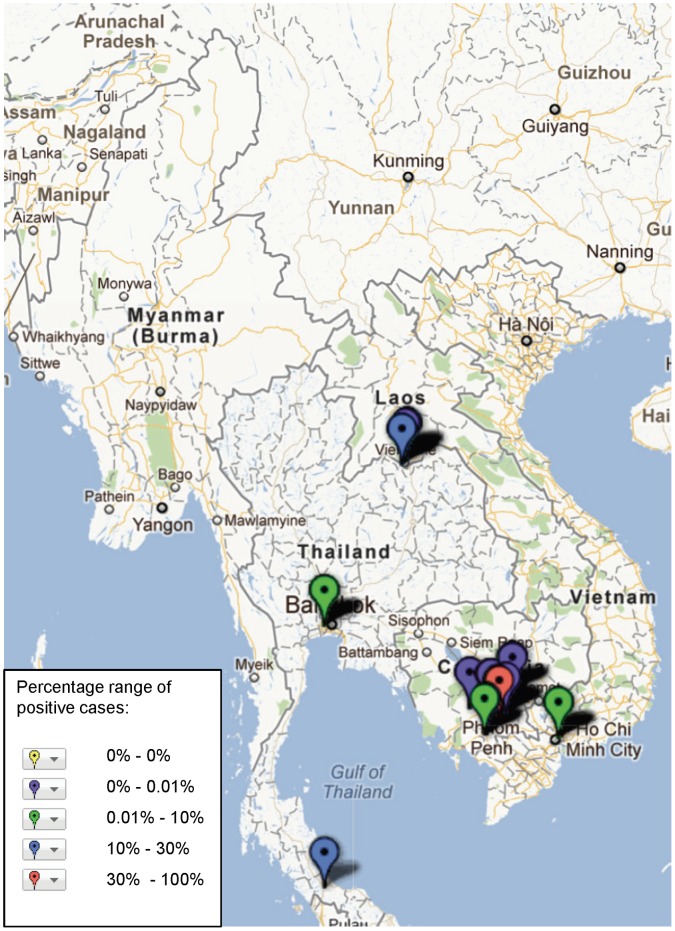
Study sites in the Mekong region where other viral infections (e.g. hepatitis viruses, herpes simplex viruses, and enterovirus) have been reported, illustrating frequency of identification (case) based on assays and sample population used in the study (see Table S1).

The geographical distribution of mapped pathogens will be influenced by investigators’ selection criteria for study sites and the methods used for sampling and pathogen detection. However, some clustering is apparent (Figures 4, 5, 6, 7, 8, 9). In particular, isolation of *S*. *typhi* and *S*. *paratyphi* is more common near the major population centres of Ho Chi Minh City and Hanoi in Viet Nam (Figure 4), while *B. pseudomallei* and *Leptospira* species were identified over a wide area including more remote border regions (Figures 5 and 6). Dengue virus is reported in studies away from major urban centres, particularly in Cambodia, Thailand and southern Viet Nam (Figure 7). It is clear that evidence for the presence or absence of a number of potentially important pathogens, particularly rickettsioses, is very limited outside of Thailand, with very little published data available retrievable in English from Myanmar and Yunnan (Figure 8). Other bacteria and other viral pathogens not specifically targeted in the literature review are grouped in Figures 10 and 11 respectively.

Variability in the methods used, especially the selection of diagnostic test, prevents a full meta-analysis to compare incidence or prevalence rates between the studies. Papers included in this mapping exercise have been published over a period of 25 years, during which laboratory methods have changed and new tests have become available. Using the acute infection aetiology papers for dengue fever reports as an example (n = 61), six different standards (previously published or commercial kits) and 13 in-house IgM/IgG ELISA methods were used. In only 36 studies was serological diagnosis confirmed by antigen or RNA detection, while in the remainder diagnosis was defined according to variable interpretations of antibody titers.

## Discussion

This review illustrates the widespread distribution of a diversity of potentially severe infections for which diagnostic tests are not routinely available or performed, and for which symptoms and signs are non-specific and potentially confused with malaria. These diseases are therefore rarely reported with routine health information system data, despite the growing importance of non-malarial fever relative to malaria [2], [4], [11], [12], [13], and management is likely to be non-specific and poor.

The pathogens recorded here are likely to represent a considerable proportion of the patients in this diagnostic gap that could benefit from early antimicrobial treatment, or for which an outbreak response may be necessary. Most can be managed effectively with accessible and relatively inexpensive drugs or specific supportive care. Failure to effectively address these alternative non-malarial aetiologies risks the health of the population and the credibility of the health service. As field malaria workers and malaria-orientated district hospitals see the majority of patients within the public sector, their inability to deal with these alternative and more common aetiologies will make it increasingly difficult to justify the continued concentration of resources to support malaria management alone, and drive patients seeking treatment away from the established health services.

A striking result of mapping these aetiological data is the identification of large areas with no information on pathogens (Figure 2) or on particular pathogens e.g. Japanese encephalitis virus (Figure 9). The majority of studies (52%) with incidence data are from Thailand, where resources for research are more readily available than some neighbouring states. Yunnan, Myanmar, Laos, outside of Vientiane, and northern Viet Nam are terra incognito for informing public health policy for fever epidemiology and management. These maps would be useful to prioritise locations of further investigations.

Limitations to interpreting the data include the great heterogeneity of published studies, including variability in study design, patient sampling and diagnostic testing. This precluded a meta-analysis. The search was also limited to English language literature. It is clear that the clustering of diseases shown here is unlikely to represent their true spatio-temporal distribution, and is likely concentrated in populations convenient to sample, such as those near a research institution. It is further restricted with regard to interpretations of change through time, but does give a guide to their presence and importance for future targeted research. Taking the above into account, these data must therefore be interpreted with caution and are not sufficient for determining empirical treatment. They should encourage and inform further targeted research to understand the aetiology of fever in SE Asia, to inform health policy.

The great diversity of tests used in the studies in this analysis may have resulted in widely varying thresholds of disease detection and precludes quantitative comparisons of incidence across sites or years. A few tests have high sensitivity and specificity, such as dengue NS1 ELISAs, but for many the true diagnostic accuracy against reference standard(s) in the field situation is unknown. There is little in the literature as to the most cost-effective forms of infectious disease laboratory capacity at different levels of health care in Asia. However, recent research suggests that, when appropriately instituted, increased diagnostic capacity could have major impact [25]. In addition to the need for accurate point of care testing for diseases such as typhus and leptospirosis, it is necessary to determine the most appropriate higher level diagnostics, and the potential impact of referral laboratory services.

There is also little previous investigation of the comparative accuracy of the diverse tests used to identify the pathogens recorded here. For example, for immunofluorescence assays for rickettsial diseases, a wide range of titre cut offs were used, usually without justification for these differences [26]. The diagnosis of acute leptospirosis remains problematic without adequate evaluation of the different serological techniques available. There have been no published comparisons of different blood culture systems in Asia and different systems were used in the papers reviewed here. Typhoid is important, especially as it can be prevented by vaccination and water supply interventions and treated relatively inexpensively. However, blood and marrow cultures are seldom available in rural southeast Asia and the Widal test is often used, despite significant problems with sensitivity and specificity [27]. While the WHO Regional Office for the Western Pacific coordinates quality assurance systems for JEV IgM ELISAs and some quality assurance systems for some other diagnostics, such systems do not exist for most diseases.

To better manage these febrile diseases, much more needs to be known about their epidemiology and health services must develop the capability to assess true disease burden. For example, as uncomplicated leptospirosis and rickettsial diseases can be effectively and safely managed by tetracycline antibiotic short courses, diagnostic tests for these diseases, or empirical doxycycline therapy, could readily improve patient outcomes [28], [29]. Although IgM RDTs are available for scrub typhus and murine typhus [30], [31], tools for leptospirosis that give an accurate and timely diagnosis are not yet readily available. Empirical management algorithms could therefore be adjusted in the meantime to reflect likely pathogens based on evidence of relative incidence in different geographical areas. However, assurance of the accuracy and safety of such algorithms will generally require much more disease-specific data of prevalence, incidence and human, environmental and geographical risk. Gathering this will require either routine use of low-cost field diagnostics coupled with high quality reporting, or the development of field-adapted screening tools that enable wide-scale surveys of pathogen prevalence, and in some cases antimicrobial sensitivity. While the data gathered here is the best available from the limited literature in this area, it highlights the need for standardization of studies, including clinical definitions and pathological methods to provide the data necessary for improving clinical management on a wide scale.

A necessary aspect of the development of national empirical treatment guidelines is within-region diversity of disease aetiology and pathogen antibiotic susceptibility patterns. Modeling suggests that spatially stratified empirical treatment protocols for undifferentiated fevers will have a higher impact than national protocols if there is significant within country diversity in fever aetiology and pathogen antibiotic resistance patterns. This therefore suggests that the causes of non-malarial fever should be investigated at multiple locations in a region to optimize empirical treatment [25].

While data on the allocation of research and development funding of these diseases is scanty, the case of malaria funding suggests that current funding for diagnostics will be minimal [32]. Better standardization of tests and techniques is needed, together with analytical methods that enable true comparisons of disease rates. Systems to share patient data, samples and research protocols should be promoted. Data sharing is yet not widely adopted by the clinical research community but will be critically important to construct a more comprehensive and intelligent source of information. Strict ethical principles and ways to protect ownership of primary data contributors will also be key to ensure the success of such models. The feasibility of such approaches, demonstrated in the collection, mapping and dissemination of antimalarial resistance data by the WorldWide Antimalarial Resistance Network (WWARN) [33], is illustrated here for the development of global maps of distribution of major pathogens of non-malarial febrile illness that could then be enhanced by the targeted collection of prospective data. Mobilizing sufficient resources will require persuasive data and clear arguments regarding the most likely routes through which available funds can produce impact. In contrast to drug and vaccine development, arguments for investment in diagnostics can be based on far lower development costs and higher probability of successful product development in a relatively short time-frame [32].

### Conclusion

This mapping demonstrates a very heterogeneous distribution of information on the causes of fever in the Mekong countries. Expanding the maps of pathogen distribution is rapidly achievable at relatively low cost and would provide a useful starting point both to identify where pathogens are likely to be encountered, and where focused research could most efficiently address the major knowledge gaps. Standardization of study methodology and expansion of the database could then guide the development of improved algorithms for syndromic management of fever, prioritize diagnostic development, and potentially guide empirical therapy.

## Supporting Information

Table S1 **Evidence for the frequency of acute infections with pathogens known to cause fever in the Mekong region: location, age groups, patient admission status, the main clinical inclusion criteria, sample type, and laboratory tests of studies from 1988 to 2011.**
(DOCX)

Table S2 **PubMed-Medline references for all papers of pathogen identification listed in Table S1.**
(DOCX)

Table S3 **Frequency of past infection with pathogen known to cause fever in the Mekong region: location, sample type, and laboratory tests of studies from 1991 to 2010.**
(DOCX)
