# Supplementary material for: Mapping the Aetiology of Non-Malarial Febrile Illness in Southeast Asia through a Systematic Review—Terra Incognita Impairing Treatment Policies
Source: PLoS One. 2012 Sep 6;7(9):e44269. doi: 10.1371/journal.pone.0044269 (PMC3435412; doi:10.1371/journal.pone.0044269)
Supplement: Table S1 — Evidence for the frequency of acute infections with pathogens known to cause fever in the Mekong region: location, age groups, patient admission status, the main clinical inclusion criteria, sample type, and laboratory tests of studies from 1988 to 2011. (DOCX) [file pone.0044269.s001.docx]

**Table S1.** Evidence for the frequency of acute infections with pathogens causing fever in the Mekong region: location, age groups, patient admission status, the main clinical inclusion criteria, sample type, and laboratory tests of studies from 1988 to 2011.

| **Location(s)** | | **Pathogens (date)** | | **Age groups (y: years)** | | **Inpatient / Outpatient/ Population survey** | | | **Study clinical inclusion criteria^1^** | | | **Positive / Tested samples (%)** | | | **Sample(s) / Test(s) used^2^** | | | **Reference** | | |  |
| --- | --- | --- | --- | --- | --- | --- | --- | --- | --- | --- | --- | --- | --- | --- | --- | --- | --- | --- | --- | --- | --- |
| **THAILAND** | | | | | | | | | | | | | | | | | | | | |  |
| Prachin Buri | | *Rickettsia typhi* | ≥15 y | | | Inpatient | | | ≥4 days of fever, negative malaria smears | | | 14/19 (73.7) | Blood/IFA | | | | | | | Brown A. *et al.* 1988 |  |
|  | | *Orientia tsutsugamushi* |  | | |  | | |  | | | 2/19 (10.5) | Blood/IFA | | | | | | |  |  |
|  | | *Rickettsia sibirica* |  | | |  | | |  | | | 0/19 (0.0) | Blood/IFA | | | | | | |  |  |
| Ubon Ratchathani | | *Burkholderia pseudomallei* | All ages | | | Inpatient | | | Short history of fever, suspected septicemia | | | 63/255 (24.7) | Blood/Culture | | | | | | | Chaowagul W. *et al.* 1989 |  |
|  | | *Escherichia coli* |  | | |  | | |  | | | 55/255 (21.6) | Blood/Culture | | | | | | |  |  |
|  | | Staphylococci |  | | |  | | |  | | | 53/255 (20.8) | Blood/Culture | | | | | | |  |  |
|  | | *Enterobacter* spp |  | | |  | | |  | | | 42/255 (16.5) | Blood/Culture | | | | | | |  |  |
|  | | *Pseudomonas aeruginosa* |  | | |  | | |  | | | 22/255 (8.6) | Blood/Culture | | | | | | |  |  |
|  | | Streptococci |  | | |  | | |  | | | 21/255 (8.2) | Blood/Culture | | | | | | |  |  |
|  | | *Streptococcus pneumonia* |  | | |  | | |  | | | 14/255 (5.5) | Blood/Culture | | | | | | |  |  |
|  | | *Pseudomonas* |  | | |  | | |  | | | 7/255 (2.8) | Blood/Culture | | | | | | |  |  |
|  | | *Aeromonas* spp |  | | |  | | |  | | | 2/255 (0.8) | Blood/Culture | | | | | | |  |  |
|  | | *Acinetobacter* spp |  | | |  | | |  | | | 1/255 (0.4) | Blood/Culture | | | | | | |  |  |
|  | | *Pseudomonas cepacia* |  | | |  | | |  | | | 1/255 (0.4) | Blood/Culture | | | | | | |  |  |
|  | | *Salmonella* spp |  | | |  | | |  | | | 1/255 (0.4) | Blood/Culture | | | | | | |  |  |
|  | | *Shigella* ssp |  | | |  | | |  | | | 1/255 (0.4) | Blood/Culture | | | | | | |  |  |
| Prachin Buri | | *Rickettsia typhi* | All ages | | | Inpatient | | | documented fever (≥38°C) and at least headache and/or myalgia and/or lassitude and/or anorexia | | | 26/37 (70.3) | Blood/Indirect immunoperoxidase assay for rickettsial IgM/IgG | | | | | | | Duffy P. *et al.* 1990 |  |
|  | | *Orientia tsutsugamushi* |  | | |  | | |  | | | 2/37 (5.4) | Blood/Indirect immunoperoxidase assay for rickettsial IgM/IgG | | | | | | |  |  |
| Songkhla | | *Orientia tsutsugamushi* | 2-14 y | | | Inpatient | | | >6 days of fever (≥38.5°C) | | | 18/320 (5.6) | Blood/Weil-Felix test | | | | | | | Silpapojakul K. *et al.* 1991 |  |
|  | | *Rickettsia typhi* |  | | |  | | |  | | | 10/320 (3.1) | Blood/Weil-Felix test | | | | | | |  |  |
|  | | *Leptospira* |  | | |  | | |  | | | 10/320 (3.1) | Blood/IFA | | | | | | |  |  |
| Prachin Buri | | *Orientia tsutsugamushi* | 16-69 y | | | Inpatient | | | >4 days of fever (>38°C), 3 negative malaria smears | | | 39/67 (58.2) | Blood/Indirect immunoperoxidase assay for rickettsial IgM/IgG | | | | | | | Wilde H. *et al.* 1991 |  |
|  | | *Rickettsia typhi* |  | | |  | | |  | | | 6/67 (9.0) | Blood/Indirect immunoperoxidase assay for rickettsial IgM/IgG | | | | | | |  |  |
|  | | Dengue virus |  | | |  | | |  | | | 4/67 (6.0) | Blood/IgM/IgG ELISA (Innis), haemagglutination inhibition assay | | | | | | |  |  |
| Ubon Ratchathani | | *Burkholderia pseudomallei* | 0-18 y | | | Inpatient | | | N/A | | | 17/3528 (0.5) | Blood/Indirect haemagglutination assay, Culture | | | | | | | Kanaphun P. *et al.* 1993 |  |
| Chiang Mai | | Spotted fever group rickettsia | 12, 28 and 47 y | | | Inpatient | | | ≤10-day history of fever, myalgia and headache | | | 3/3 (100.0) | Blood/Rickettsial IgM/IgG ELISA, IFA, indirect immunoperoxidase assay for rickettsial IgM/IgG, Weil-Felix test | | | | | | | Sirisanthana T. *et al.* 1994 |  |
| Nonthaburi | | *Orientia tsutsugamushi* | 0-80 y | | | Population survey (asymptomatic)^⧫^ | | | N/A | | | 45/215 (20.9) | Blood/Indirect immunoperoxidase assay for rickettsial IgM/IgG | | | | | | | Strickman D. *et al.* 1994 |  |
|  | | *Rickettsia typhi* |  | | |  | | |  | | | 18/215 (8.4) | Blood/Indirect immunoperoxidase assay for rickettsial IgM/IgG | | | | | | |  |  |
|  | | Spotted fever group rickettsia |  | | |  | | |  | | | 9/215 (4.2) | Blood/Indirect immunoperoxidase assay for rickettsial IgM/IgG | | | | | | |  |  |
| Ubon Ratchathani | | *Burkholderia pseudomallei* | <1-86 y | | | Inpatient | | | Suspected melioidosis infection | | | 4/100000 (0.0) | Blood/Indirect haemagglutination assay | | | | | | | Suputtamongkol Y. *et al.* 1994 |  |
| Songkhla | | *Leptospira* | All ages | | | Inpatient | | | History of fever, suspected leptospirosis | | | 58/175 (33.1) | Blood/MAT, IFA, Culture | | | | | | | Appassakij H. *et al.* 1995 |  |
|  | | Dengue virus |  | | |  | | |  | | | 27/93 (29.0) | Blood/ Haemagglutination inhibition assay | | | | | | |  |  |
|  | | Gram-negative bacteremia |  | | |  | | |  | | | 27/93 (29.0) | Blood/Culture | | | | | | |  |  |
|  | | *Orientia tsutsugamushi* |  | | |  | | |  | | | 15/93 (16.1) | Blood/IFA | | | | | | |  |  |
|  | | Hepatitis virus B |  | | |  | | |  | | | 12/93 (12.9) | Blood/Anti-HBsAg IgM ELISA | | | | | | |  |  |
|  | | *Rickettsia typhi* |  | | |  | | |  | | | 12/93 (12.9) | Blood/IFA | | | | | | |  |  |
| Ubon Ratchathani | | *Burkholderia pseudomallei* | 17-78 y | | | Inpatient | | | Suspected septicemia | | | 108/418 (25.8) | Blood/Culture | | | | | | | Walsh A. *et al.* 1995 |  |
|  | | Gram-negative bacilli |  | | |  | | |  | | | 13/418 (3.1) | Blood/Culture | | | | | | |  |  |
|  | | Gram-positive cocci |  | | |  | | |  | | | 3/418 (0.7) | Blood/Culture | | | | | | |  |  |
| Kanchanaburi | | *Orientia tsutsugamushi* | 18-50 y | | | Population survey (asymptomatic)^⧫^ | | | N/A | | | 8/55 (14.6) | Blood/IFA | | | | | | | Eamsila C. *et al.* 1996 |  |
| Prachin Buri | | *Orientia tsutsugamushi* |  | | |  | | |  | | | 7/76 (9.2) | Blood/IFA | | | | | | |  |  |
| Ubon Ratchathani | | *Orientia tsutsugamushi* |  | | |  | | |  | | | 12/223 (5.4) | Blood/IFA | | | | | | |  |  |
| Phitsanulok | | *Orientia tsutsugamushi* |  | | |  | | |  | | | 12/293 (4.1) | Blood/IFA | | | | | | |  |  |
| Buri Ram | | *Orientia tsutsugamushi* |  | | |  | | |  | | | 4/121 (3.3) | Blood/IFA | | | | | | |  |  |
| Tak | | *Orientia tsutsugamushi* |  | | |  | | |  | | | 2/92 (2.2) | Blood/IFA | | | | | | |  |  |
| Uttaradit | | *Orientia tsutsugamushi* |  | | |  | | |  | | | 0/51 (0.0) | Blood/IFA | | | | | | |  |  |
| Sisaket | | *Orientia tsutsugamushi*: | 18-50 y | | | Population survey (asymptomatic)^⧫^ | | | N/A | | |  |  | | | | | | | Frances S. *et al.* 1997 |  |
|  | | January 1992 |  | | |  | | |  | | | 9/214 (4.2) | Blood/IFA | | | | | | |  |  |
|  | | April 1992 |  | | |  | | |  | | | 0/193 (0.0) | Blood/IFA | | | | | | |  |  |
|  | | September 1992 |  | | |  | | |  | | | 8/213 (3.8) | Blood/IFA | | | | | | |  |  |
| Bangkok, Kamphaeng Phet | | Dengue virus | 0.6-14 y | | | Inpatient | | | <3 days of fever | | | 59/60 (98.3) | Blood/Viral isolation | | | | | | | Vaughn D. *et al.* 1997 |  |
|  | | Dengue virus |  | | |  | | |  | | | 60/189 (31.8) | Blood/IgM/IgG ELISA (Innis), haemagglutination inhibition assay | | | | | | |  |  |
|  | | *Salmonella*, *Haemophilus influenzae* |  | | |  | | |  | | | 3/189 (1.6) | Blood/Culture | | | | | | |  |  |
| Chiang Rai | | *Orientia tsutsugamushi* | All ages | | | Not specified | | | ≥2 days of fever | | | 35/126 (27.8) | Blood/Indirect immunoperoxidase assay for rickettsial IgM/IgG, Dip-s-Ticks | | | | | | | Watt G. *et al.* 1998 |  |
| Nonthaburi | | Dengue virus | 4-13 y | | | Population survey (asymptomatic)^⧫^ | | | N/A | | | 33/1000 (3.3) | Blood/IgM ELISA (Innis) | | | | | | | Strickman D. *et al.* 2000 |  |
|  | | Japanese encephalitis virus |  | | |  | | |  | | | 7/1000 (0.7) | Blood/JE IgM ELISA | | | | | | |  |  |
| Chachoen-gsao | | Dengue virus |  | | |  | | |  | | | 95/1000 (9.5) | Blood/ IgM ELISA (Innis) | | | | | | |  |  |
|  | | Japanese encephalitis virus |  | | |  | | |  | | | 32/1000 (3.2) | Blood/JE IgM ELISA | | | | | | |  |  |
| Nakhon Ratchasima | | *Leptospira* | 15-59 y | | | Not specified | | | History of fever, headache, and myalgia | | | 59/169 (34.9) | Blood/*Leptospira* IgM ELISA (PanBio) | | | | | | | Tangkanakul W. *et al.* 2000 |  |
| Bangkok**,** Kamphaeng Phet | | Dengue virus | 0.5-14 y | | | Inpatient | | | History of fever for <72 h | | | 165/168 (98.2) | Blood/RT-PCR | | | | | | | Vaughn D. *et al.* 2000 |  |
|  | | Dengue virus |  | | |  | | |  | | | 162/168 (96.4) | Blood/Viral isolation | | | | | | |  |  |
|  | | Dengue virus |  | | |  | | |  | | | 168/456 (36.8) | Blood/IgM/IgG ELISA (Innis), haemagglutination inhibition assay | | | | | | |  |  |
| Bangkok | | Dengue virus | 0-13 y | | | Inpatient | | | History of fever | | | 8/40 (20.0) | Blood/IgM/IgG ELISA (Innis), haemagglutination inhibition assay | | | | | | | Chokephaibulkit K. *et al.* 2001 |  |
|  | | Dengue virus |  | | |  | | |  | | | 7/40 (17.5) | Blood/Viral isolation, RT-PCR | | | | | | |  |  |
|  | | Japanese encephalitis virus |  | | |  | | |  | | | 6/40 (15.0) | Blood/ JE IgM/IgG ELISA | | | | | | |  |  |
|  | | Herpes simplex virus |  | | |  | | |  | | | 4/40 (10.0) | Blood/IgM/IgG ELISA, PCR | | | | | | |  |  |
|  | | Human Herpes virus type 6 |  | | |  | | |  | | | 3/40 (7.5) | Blood/IgM/IgG ELISA, PCR | | | | | | |  |  |
|  | | Mumps virus |  | | |  | | |  | | | 2/40 (5.0) | Blood/IgM/IgG ELISA, PCR | | | | | | |  |  |
|  | | Enterovirus |  | | |  | | |  | | | 1/40 (2.5) | Blood/PCR | | | | | | |  |  |
| Bangkok | Dengue virus | | All ages | | | | | Inpatient | Suspected dengue infection | | | 664/949 (70.0) | Blood/IgM blot (Genelabs), IgM rapid test (PanBio), haemagglutination assay | | | | | | | Kowitdamrong E. *et al.* 2001 |  |
| Bangkok | Dengue virus | | 0-15 y | | | | | Inpatient | History of fever | | | 139/996 (14.0) | Blood/IgM/IgG ELISA (in-house), haemagglutination inhibition assay | | | | | | | Pancharoen C. *et al.* 2001 |  |
| Kamphaeng Phet | Dengue virus: | | 6-12 y | | | | | Population survey (symptomatic) | History of fever ≤7 days | | |  | Blood/IgM/IgG ELISA (Innis), haemagglutination inhibition assay, viral isolation, RT-PCR | | | | | | | Endy T. *et al.* 2002 |  |
|  | (1998) | |  | | | | |  |  | | | 168/2119 (8.0) |  | | | | | | |  |  |
|  | (1999) | |  | | | | |  |  | | | 126/1928 (6.5) |  | | | | | | |  |  |
|  | (2000) | |  | | | | |  |  | | | 27/1713 (1.6) |  | | | | | | |  |  |
| Bangkok | Dengue virus | | <1 y | | | | | Inpatient | Suspected dengue infection | | | 237/4872 (4.9) | Blood/IgM/IgG ELISA (AFRIMS), haemagglutination inhibition assay | | | | | | | Halstead S. *et al.* 2002 |  |
|  | Dengue virus | |  | | | | |  |  | | | 114/237 (48.1) | Viral isolation | | | | | | |  |  |
| Chonburi | Dengue virus | | ≤15 y | | | | | Inpatient | History of fever | | | 20/983 (2.0) | Blood/IgM/IgG ELISA (AFRIMS), haemagglutination inhibition assay | | | | | | | Hongsiriwon S. *et al.* 2002 |  |
| Ang Thong | Dengue virus | | All ages | | | | | Inpatient | N/A | | | 52/92 (56.5) | Blood/IgM/IgG rapid test (PanBio), haemagglutination inhibition assay | | | | | | | Kittigul L. *et al.* 2002 |  |
| Loei | *Leptospira* | | 8-76 y | | | | | Inpatient | History of fever, headache, and myalgia | | | 148/475 (31.2) | Blood/MAT | | | | | | | Niwattayakul K. *et al.* 2002 |  |
| Buri Ram | *Leptospira* | | 15-65 y | | | | | Population survey (a-and symptomatic) | N/A | | | 43/104 (41.4) | Blood/Lepto-Dipstick Test | | | | | | | Phraisuwan P. *et al.* 2002 |  |
| Ang Thong | Dengue virus | | <15 y | | | | | Population survey (symptomatic) | Suspected dengue infection | | | 62/167 (37.1) | Blood/IgM/IgG ELISA (Innis), haemagglutination inhibition assay | | | | | | | Kittigul L. *et al.* 2003 |  |
|  | Dengue virus | |  | | | | |  |  | | | 14/41 (34.2) | Blood/Viral isolation | | | | | | |  |  |
| Bangkok | Dengue virus: | | <18 y | | | | | Inpatient | Suspected dengue infection | | |  | Blood/IgM/IgG ELISA (in-house), haemagglutination inhibition assay, plaque reduction neutralization assay, viral isolation, RT-PCR (test differed over time). | | | | | | | Nisalak A. *et al.* 2003 |  |
|  | (1973) | |  | | | | |  |  | | | 88/131 (67.2) |  | | | | | | |  |  |
|  | (1974) | |  | | | | |  |  | | | 109/142 (76.8) |  | | | | | | |  |  |
|  | (1975) | |  | | | | |  |  | | | 309/385 (80.3) |  | | | | | | |  |  |
|  | (1976) | |  | | | | |  |  | | | 147/171 (86.0) |  | | | | | | |  |  |
|  | (1977) | |  | | | | |  |  | | | 419/486 (86.2) |  | | | | | | |  |  |
|  | (1978) | |  | | | | |  |  | | | 128/174 (73.6) |  | | | | | | |  |  |
|  | (1979) | |  | | | | |  |  | | | 237/281 (84.3) |  | | | | | | |  |  |
|  | (1980) | |  | | | | |  |  | | | 621/717 (86.6) |  | | | | | | |  |  |
|  | (1981) | |  | | | | |  |  | | | 169/184 (91.9) |  | | | | | | |  |  |
|  | (1982) | |  | | | | |  |  | | | 165/185 (89.2) |  | | | | | | |  |  |
|  | (1983) | |  | | | | |  |  | | | 348/380 (91.6) |  | | | | | | |  |  |
|  | (1984) | |  | | | | |  |  | | | 631/707 (89.3) |  | | | | | | |  |  |
|  | (1985) | |  | | | | |  |  | | | 474/531 (89.3) |  | | | | | | |  |  |
|  | (1986) | |  | | | | |  |  | | | 517/577 (89.6) |  | | | | | | |  |  |
|  | (1987) | |  | | | | |  |  | | | 1354/1457 (92.9) |  | | | | | | |  |  |
|  | (1988) | |  | | | | |  |  | | | 394/477 (82.6) |  | | | | | | |  |  |
|  | (1989) | |  | | | | |  |  | | | 1082/1190 (90.9) |  | | | | | | |  |  |
|  | (1990) | |  | | | | |  |  | | | 976/1143 (85.4) |  | | | | | | |  |  |
|  | (1991) | |  | | | | |  |  | | | 346/538 (64.3) |  | | | | | | |  |  |
|  | (1992) | |  | | | | |  |  | | | 382/471 (81.1) |  | | | | | | |  |  |
|  | (1993) | |  | | | | |  |  | | | 677/774 (87.5) |  | | | | | | |  |  |
|  | (1994) | |  | | | | |  |  | | | 468/584 (80.1) |  | | | | | | |  |  |
|  | (1995) | |  | | | | |  |  | | | 513/624 (82.2) |  | | | | | | |  |  |
|  | (1996) | |  | | | | |  |  | | | 497/631 (78.8) |  | | | | | | |  |  |
|  | (1997) | |  | | | | |  |  | | | 1488/1690 (88.1) |  | | | | | | |  |  |
|  | (1998) | |  | | | | |  |  | | | 1368/1668 (82.0) |  | | | | | | |  |  |
|  | (1999) | |  | | | | |  |  | | | 696/1079 (64.5) |  | | | | | | |  |  |
| Kanchanaburi | Spotted fever group rickettsia | | ≥20 y | | | | | Not specified | ≤2 days of fever | | | 8/46 (17.4) | Blood/IgM ELISA, IFA, Dot-ELISA (total Ig), IFA | | | | | | | Parola P. *et al.* 2003 |  |
|  | *Rickettsia typhi* | |  | | | | |  |  | | | 4/46 (8.7) | Blood/IFA | | | | | | |  |  |
|  | *Orientia tsutsugamushi* | |  | | | | |  |  | | | 3/46 (6.5) | Blood/IFA | | | | | | |  |  |
|  | *Coxiella burnetii* | |  | | | | |  |  | | | 0/46 (0.0) | Blood/IFA | | | | | | |  |  |
|  | *Ehrlichia chaffeensis* | |  | | | | |  |  | | | 0/46 (0.0) | Blood/IFA | | | | | | |  |  |
| Songkhla | Dengue virus | | 0-15 y | | | | | Inpatient | Acute fever (>38°C) >1 day | | | 53/180 (29.4) | Blood/ Haemagglutination inhibition assay | | | | | | | Pradutkanchana J. *et al.* 2003 |  |
|  | *Leptospira* | |  | | | | |  |  | | | 49/180 (27.2) | Blood/IFA | | | | | | |  |  |
|  | *Orientia tsutsugamushi* | |  | | | | |  |  | | | 2/180 (1.1) | Blood/IFA | | | | | | |  |  |
|  | *Rickettsia sibirica* | |  | | | | |  |  | | | 0/180 (0.0) | Blood/IFA | | | | | | |  |  |
|  | *Rickettsia typhi* | |  | | | | |  |  | | | 0/180 (0.0) | Blood/IFA | | | | | | |  |  |
| Phrae | *Leptospira* | | 15-100 y | | | | | Inpatient | N/A | | | 295/362 (81.5) | Blood/MAT,IFA, Lepto-Dipstick Test | | | | | | | Tantitanawat S. *et al.* 2003 |  |
| Nakhon Ratchasima | Dengue virus | | 17-33 y | | | | | Outpatient | History of fever ≤2 weeks | | | 35/156 (22.4) | Blood/IgM ELISA (in-house) | | | | | | | Watt G. *et al.* 2003 |  |
|  | Japanese encephalitis virus | |  | | | | |  |  | | | 22/156 (14.1) | Blood/JE IgM capture ELISA | | | | | | |  |  |
| Songkhla | *Leptospira* | | All ages | | | | | Inpatient | Acute fever (>38°C) >1day | | | 124/348 (35.6) | Blood/MAT, IFA, latex agglutination test, Lepto dipstick Test | | | | | | | Kemapunmanus M. *et al.* 2004 |  |
| Khon Kaen | *Burkholderia pseudomallei* | | ≥15 y | | | | | Not specified | Suspected melioidosis infection | | | 68/162 (42.0) | Blood/Indirect haemagglutination assay, Culture | | | | | | | Reechaipichitkul W. 2004 |  |
| Nakhon Ratchasima | *Leptospira* | | 12-50 y | | | | | Inpatient | N/A | | | 96/343 (28.0) | Blood/MAT | | | | | | | Tansuphasiri U. *et al.* 2004 |  |
| Nakhon Ratchasima, Loei, Buri Ram, Udon Thani | *Leptospira* | | 13-92 y | | | | | Inpatient | Acute fever <15 days | | | 264/540 (48.9) | Blood/MAT, IFA, MCAT, Culture | | | | | | | Suputtamongkol Y. *et al.* 2004 |  |
| Chonburi | Dengue virus | | 0.3-66 y | | | | | Inpatient | Suspected dengue infection | | | 128/347 (36.9) | Blood/IgM/IgG ELISA (AFRIMS), haemagglutination inhibition assay | | | | | | | Wichmann O. *et al.* 2004 |  |
| Songkhla, Bangkok, Nakhon Ratchasima, Ratchaburi, Lampang, Pathum Thani | Dengue virus | | <1-66 y | | | | | Population survey (symptomatic) | Suspected dengue infection | | | 2715/5980 (45.4) | Blood/IgM/IgG ELISA (Innis), viral isolation, RT-PCR | | | | | | | Anantapreecha S. *et al.* 2005 |  |
| Ubon Ratchathani | *Burkholderia pseudomallei* | | All ages | | | | | Inpatient | Suspected melioidosis infection | | | 199/324 (61.4) | Throat swab/Culture | | | | | | | Huis In't Veld D. *et al.* 2005 |  |
|  |  | |  | | | | |  |  | | | 444/726 (61.2) | Sputum/Culture | | | | | | |  |  |
|  |  | |  | | | | |  |  | | | 386/700 (55.1) | Blood/Culture | | | | | | |  |  |
| Ubon Ratchathani | *Burkholderia pseudomallei* | | 1-89 y | | | | | Inpatient | Suspected melioidosis infection | | | 487/727 (67.0) | Blood/Culture | | | | | | | Limmathurotsakul D. *et al.* 2005 |  |
| Loei | *Leptospira* | | All ages | | | | | In-and outpatient | Suspected leptospirosis infection | | | 52/98 (53.1) | Blood/MAT, Culture | | | | | | | Niwetpathomwat A. *et al.* 2005 |  |
| Khon Kaen | Dengue virus | | 1-14 y | | | | | Inpatient | Acute fever >37.8°C for 6-21 days | | | 7/25 (28.0) | Blood/IgM/IgG ELISA (Innis) | | | | | | | Sripanidkulchai R. *et al.* 2005 |  |
|  | *Leptospira* | |  | | | | |  |  | | | 2/25 (8.0) | Blood/MAT, IFA | | | | | | |  |  |
|  | Japanese encephalitis virus | |  | | | | |  |  | | | 0/25 (0.0) | Blood/JE IgM/IgG ELISA | | | | | | |  |  |
|  | *Orientia tsutsugamushi* | |  | | | | |  |  | | | 0/25 (0.0) | Blood/IFA | | | | | | |  |  |
|  | *Rickettsia typhi* | |  | | | | |  |  | | | 0/25 (0.0) | Blood/IFA | | | | | | |  |  |
| Chiang Mai, Lamphun | Dengue virus | | All ages | | | | | Population survey (asymptomatic)^⧫^ | N/A | | | 113/1750 (6.5) | Blood/IgM ELISA (Focus) | | | | | | | Van Benthem B. *et al.* 2005 |  |
| Ubon Ratchathani | *Burkholderia pseudomallei* | | All ages | | | | | Inpatient | Suspected melioidosis infection | | | 84/120 (70.0) | Blood/Pus/Urine/ Culture | | | | | | | Wuthiekanun V. *et al.* 2005 |  |
| Kanchanaburi | *Leptospira* | | 20-87 y | | | | | In-and outpatient | >2 days of fever | | | 107/613 (17.5) | Blood/*Leptospira* IgM ELISA, MAT | | | | | | | Ellis R. *et al.* 2006 |  |
|  | *Plasmodium falciparum* | |  | | | | |  |  | | | 95/613 (15.5) | Blood/Blood smear Giemsa microscopy | | | | | | |  |  |
|  | *Plasmodium vivax* | |  | | | | |  |  | | | 48/613 (7.8) | Blood/Blood smear Giemsa microscopy | | | | | | |  |  |
|  | Spotted fever group rickettsia | |  | | | | |  |  | | | 20/613 (3.3) | Blood/IFA | | | | | | |  |  |
|  | Dengue virus | |  | | | | |  |  | | | 9/613 (1.5) | Blood/IgM/IgG ELISA (AFRIMS), RT-PCR | | | | | | |  |  |
|  | *Rickettsia typhi* | |  | | | | |  |  | | | 9/613 (1.5) | Blood/ IFA | | | | | | |  |  |
|  | *Orientia tsutsugamushi* | |  | | | | |  |  | | | 7/613 (1.1) | Blood/ IFA | | | | | | |  |  |
|  | *S*. *enterica* serovar Typhi | |  | | | | |  |  | | | 5/613 (0.8) | Blood/RT-PCR | | | | | | |  |  |
|  | Japanese encephalitis virus | |  | | | | |  |  | | | 1/613 (0.2) | Blood/JE IgM/IgG ELISA | | | | | | |  |  |
| Nakhon Pathom | Dengue virus | | 0->50 y | | | | | Population survey (asymptomatic)^⧫^ | N/A | | | 29/329 (8.8) | Blood/IgM/IgG ELISA (Innis), RT-PCR | | | | | | | Poblap T. *et al.* 2006 |  |
|  | Japanese encephalitis virus | |  | | | | |  |  | | | 1/329 (0.3) | Blood/JE IgM/IgG MAC ELISA | | | | | | |  |  |
| Kanchanaburi, Tak | *Plasmodium falciparum* | | 15-87 y | | | | | Population survey (symptomatic) | N/A | | | 194/194 (100.0) | Blood/Blood smear Giemsa microscopy | | | | | | | Singhsilarak T. *et al.* 2006 |  |
|  | *Rickettsia typhi* | |  | | | | |  |  | | | 45/194 (23.2) | Blood/IFA | | | | | | |  |  |
|  | *Orientia tsutsugamushi* | |  | | | | |  |  | | | 29/194 (15.0) | Blood/IFA | | | | | | |  |  |
|  | *Leptospira* | |  | | | | |  |  | | | 15/194 (7.7) | Blood/IFA | | | | | | |  |  |
|  | Dengue virus | |  | | | | |  |  | | | 0/29 (0.0) | Blood/IgM/IgG ELISA (Innis) | | | | | | |  |  |
| Udon Thani | *Orientia tsutsugamushi* | | ≥15 y | | | | | Inpatient | History of fever (>37.8°C) | | | 82/183 (44.8) | Blood/IFA | | | | | | | Sonthayanon P. *et al.* 2006 |  |
|  | *Orientia tsutsugamushi* | |  | | | | |  |  | | | 156/722 (21.6) | Blood/ PCR | | | | | | |  |  |
| Nakhon Ratchasima | *Leptospira* | | 15-87 y | | | | | Not specified | Acute fever <15 days | | | 61/171 (35.7) | Blood/MAT, IFA, Culture | | | | | | | Suttinont C. *et al.* 2006 |  |
|  | *Orientia tsutsugamushi* | |  | | | | |  |  | | | 40/171 (23.4) | Blood/Micro-immunofluorescence assay | | | | | | |  |  |
|  | Dengue and/or Influenza | |  | | | | |  |  | | | 9/171 (5.3) | Blood/IgM/IgG ELISA (Ward Medic) | | | | | | |  |  |
|  | *R*. *typhi* and *R*. *helvetica* | |  | | | | |  |  | | | 3/171 (1.8) | Blood/Micro-immunofluorescence assay | | | | | | |  |  |
| Loei | *Leptospira* | |  | | | | |  |  | | | 94/197 (47.7) | Blood/IFA, MAT, Culture | | | | | | |  |  |
|  | Dengue and/or Influenza | |  | | | | |  |  | | | 24/197 (12.2) | Blood/IgM/IgG ELISA (Ward Medic) | | | | | | |  |  |
|  | *Orientia tsutsugamushi* | |  | | | | |  |  | | | 9/197 (4.6) | Blood/Micro-immunofluorescen ce assay | | | | | | |  |  |
|  | *R*. *typhi* and *R*. *helvetica* | |  | | | | |  |  | | | 8/197 (4.1) | Blood/Micro-immunofluorescen ce assay | | | | | | |  |  |
| Buri Ram | *Leptospira* | |  | | | | |  |  | | | 92/296 (31.1) | Blood/IFA, MAT, Culture | | | | | | |  |  |
|  | *Orientia tsutsugamushi* | |  | | | | |  |  | | | 62/296 (21.0) | Blood/Micro-immunofluorescen ce assay | | | | | | |  |  |
|  | Dengue and/or Influenza | |  | | | | |  |  | | | 21/296 (7.1) | Blood/ IgM/IgG ELISA (Ward Medic) | | | | | | |  |  |
|  | *R*. *typhi* and *R*. *helvetica* | |  | | | | |  |  | | | 14/296 (4.7) | Blood/MAT, IFA, Culture | | | | | | |  |  |
| Ratchaburi | Dengue and/or Influenza | |  | | | | |  |  | | | 21/93 (22.6) | Blood/IgM/IgG ELISA (Ward Medic) | | | | | | |  |  |
|  | *Orientia tsutsugamushi* | |  | | | | |  |  | | | 19/93 (20.4) | Blood/Micro- immunofluorescence assay | | | | | | |  |  |
|  | *Leptospira* | |  | | | | |  |  | | | 6/93 (6.5) | Blood/IFA, MAT, Culture | | | | | | |  |  |
|  | *R*. *typhi* and *R*. *helvetica* | |  | | | | |  |  | | | 2/93 (2.2) | Blood/MAT, IFA, Culture | | | | | | |  |  |
| Chumphon | *Leptospira* | |  | | | | |  |  | | | 18/88 (20.5) | Blood/MAT, IFA, Culture | | | | | | |  |  |
|  | *Orientia tsutsugamushi* | |  | | | | |  |  | | | 12/88 (13.6) | Blood/Micro-immunofluorescence assay | | | | | | |  |  |
|  | Dengue and/or Influenza | |  | | | | |  |  | | | 8/88 (9.1) | Blood/IgM/IgG ELISA (Ward Medic) | | | | | | |  |  |
|  | *R*. *typhi* and *R*. *helvetica* | |  | | | | |  |  | | | 2/88 (2.3) | Blood/MAT, IFA, Culture | | | | | | |  |  |
| Chiang Rai, Khon Kaen | Dengue virus | | >6 y | | | | | Not specified | <2 weeks of fever (>38°C) | | | 132/704 (18.8) | Blood/IgM/IgG ELISA (AFRIMS), haemagglutination inhibition assay, IgM/IgG rapid test (PanBio) | | | | | | | Cohen A. *et al.* 2007 |  |
|  | *Leptospira* | |  | | | | |  |  | | | 67/704 (9.5) | Blood/*Leptospira* IgM ELISA, MAT | | | | | | |  |  |
| Ubon Ratchathani | *Burkholderia pseudomallei* | | All ages | | | | | Inpatient | Suspected community-acquired septicemia | | | 123/1139 (10.8) | Blood/Monoclonal antibody-based latex agglutination test, Culture | | | | | | | Ekpo P. *et al.* 2007 |  |
| Bangkok, Kamphaeng Phet | Dengue virus | | 0.5-14 y | | | | | Inpatient | History of fever | | | 350/812 (43.1) | Blood/IgM/IgG ELISA (Innis), RT-PCR | | | | | | | Libraty D. *et al.* 2007 |  |
|  | *Leptospira* | |  | | | | |  |  | | | 18/442 (4.1) | Blood/*Leptospira* IgM ELISA, MAT | | | | | | |  |  |
| Kamphaeng Phet | *Leptospira* | | 5-83 y | | | | | Inpatient | History of fever | | | 69/106 (65.1) | Blood/Lepto Dip-S-Ticks Test, *Leptospira* IgM ELISA, MAT | | | | | | | Myint K. *et al.* 2007 |  |
| Udon Thani | *Leptospira* | | ≥15 y | | | | | Inpatient | Fever (>37.8°C) | | | 115/1658 (6.9) | Blood/Cross agglutinin absorption test, Culture, PCR | | | | | | | Thaipadungpanit J. *et al.* 2007 |  |
| Udon Thani | *Leptospira* | | >14 y | | | | | Inpatient | Acute fever (<15 days) | | | 83/989 (8.4) | Blood/Culture | | | | | | | Wuthiekanun V. *et al.* 2007 |  |
| Udon Thani | *Leptospira* | | 2-95 y | | | | | Not specified | Fever, headache, muscle pain, meningism, conjunctival suffusion, and jaundice | | | 64/143 (44.8) | Blood/MAT, Culture | | | | | | | Wuthiekanun V. *et al.* 2007 |  |
| Lampang | *Leptospira* | |  | | | | |  |  | | | 28/143 (19.6) |  | | | | | | |  |  |
| Maha Sarakham | *Leptospira* | |  | | | | |  |  | | | 26/143 (18.2) |  | | | | | | |  |  |
| Rayong | *Leptospira* | |  | | | | |  |  | | | 13/143 (9.1) |  | | | | | | |  |  |
| Yasothon | *Leptospira* | |  | | | | |  |  | | | 6/143 (4.2) |  | | | | | | |  |  |
| Chainat | *Leptospira* | |  | | | | |  |  | | | 3/143 (2.1) |  | | | | | | |  |  |
| Chanthaburi | *Leptospira* | |  | | | | |  |  | | | 2/143 (1.4) |  | | | | | | |  |  |
| Prachuap Khiri Khan | *Leptospira* | |  | | | | |  |  | | | 1/143 (0.7) |  | | | | | | |  |  |
| Phatthalung | *Leptospira* | |  | | | | |  |  | | | 0/143 (0.0) |  | | | | | | |  |  |
| Bangkok | | | Dengue virus | 3-52 y | | | | | In-and outpatient | Acute fever within 5 days | | | 171/235 (72.8) | Blood/IgM/IgG ELISA (Innis), NS1 antigen (BioRad), RT-PCR, viral isolation | | | | | | | Lapphra K. *et al.* 2008 |
| Nakhon Ratchasima, Loei, Buri Ram, Udon Thani, Maha Sarakham, Yasothon | | | *Leptospira* | 13-72 y | | | | | Inpatient | Suspected leptospirosis infection | | | 149/149 (100.0) | Blood/Culture | | | | | | | Smythe L. *et al.* 2009 |
|  | | | *Leptospira* |  | | | | |  |  | | | 78/106 (73.6) | Blood/ MAT | | | | | | |  |
| Nonthaburi, Nakhon Ratchasima, Loei, Buri Ram, Udon Thani, Chumphon | | | *Orientia tsutsugamushi* | 11-92 y | | | | | Not specified | Acute fever | | | 101/141 (71.6) | Blood/PCR | | | | | | | Suputtamongkol Y. *et al.* 2009 |
|  | | | *Orientia tsutsugamushi* |  | | | | |  |  | | | 150/1663 (9.0) | Blood/ IFA | | | | | | |  |
|  | | | *Rickettsia typhi* |  | | | | |  |  | | | 21/1663 (1.3) | Blood/IFA | | | | | | |  |
| Ratchaburi | | | Dengue virus | 5-17 y | | | | | Population survey (asymptomatic and symptomatic)^⧫^ | N/A | | | 58/3547 (1.6) | Blood/IgM/IgG ELISA (Innis), plaque reduction neutralization assay, RT-PCR, viral isolation | | | | | | | Yoksan S. *et al.* 2009 |
| Ubon Ratchathani | | | *Burkholderia pseudomallei*: | All ages | | | | | Inpatient | Suspected melioidosis infection | | | Incidence rate per 100,000 people: | Blood/Sputum/Pus/ Culture | | | | | | | Limmathurotsakul D. *et al.* 2010 |
|  | | | (1997) |  | | | | |  |  | | | 11.5 |  | | | | | | |  |
|  | | | (1998) |  | | | | |  |  | | | 14.9 |  | | | | | | |  |
|  | | | (1999) |  | | | | |  |  | | | 9.8 |  | | | | | | |  |
|  | | | (2000) |  | | | | |  |  | | | 8.0 |  | | | | | | |  |
|  | | | (2001) |  | | | | |  |  | | | 8.5 |  | | | | | | |  |
|  | | | (2002) |  | | | | |  |  | | | 10,3 |  | | | | | | |  |
|  | | | (2003) |  | | | | |  |  | | | 13.0 |  | | | | | | |  |
|  | | | (2004) |  | | | | |  |  | | | 14.2 |  | | | | | | |  |
|  | | | (2005) |  | | | | |  |  | | | 15.4 |  | | | | | | |  |
|  | | | (2006) |  | | | | |  |  | | | 21.3 |  | | | | | | |  |
|  | | | Average per year: |  | | | | |  |  | | | 12.7 |  | | | | | | |  |
| Tak | | | *Plasmodium* | 15-46 y | | | | | Inpatient | Fever (>37.5°C) | | | 51/203 (25.1) | Blood/Blood smear Giemsa microscopy | | | | | | | McGready R. *et al.* 2010 |
|  | | | Dengue virus |  | | | | |  |  | | | 20/203 (9.9) | Blood/IgM ELISA (PanBio), NS1 antigen (PanBio) | | | | | | |  |
|  | | | *Orientia tsutsugamushi* |  | | | | |  |  | | | 9/203 (4.4) | Blood/IFA, RT-PCR | | | | | | |  |
|  | | | *Rickettsia typhi* |  | | | | |  |  | | | 7/203 (3.5) | Blood/IFA, RT-PCR | | | | | | |  |
|  | | | *Leptospira* |  | | | | |  |  | | | 5/203 (2.5) | Blood/MAT, Culture | | | | | | |  |
|  | | | *S*. *enterica* serovar Typhi |  | | | | |  |  | | | 2/203 (1.0) | Blood/Culture | | | | | | |  |
| Songkhla, Bangkok | | | Dengue virus | 0.5-57 y | | | | | Inpatient | Fever or hypothermia (≥38.8°C or ≤35.8°C), acute brain dysfunction, indication of lumbar puncture | | | 2/147 (1.4) | Blood/CSF/IgM ELISA (in-house), plaque reduction neutralization assay, RT-PCR | | | | | | | Olsen S. *et al.* 2010 |
|  | | | Japanese encephalitis virus |  | | | | |  |  | | | 16/147 (10.9) | Blood/JE IgM capture ELISA, plaque reduction neutralization assay | | | | | | |  |
| Bangkok | | | Dengue virus | 0.5-15 y | | | | | Inpatient | Fever (<3 days) | | | 414/1013 (40.9) | Blood/IgM/IgG ELISA (Innis), haemagglutination inhibition assay, viral isolation, RT-PCR | | | | | | | Srikiatkhachorn A. *et al.* 2010 |
| Ratchaburi | | | Dengue virus | <15 y | | | | | Population survey (asymptomatic and symptomatic)^⧫^ | History of fever | | | 25/1000 (2.5) | Blood/IgM/IgG ELISA (in-house), RT-PCR, Culture | | | | | | | Wichmann O. *et al*. 2011 |
|  | | |  |  | | | | |  |  | | |  |  | | | | | | |  |
| Kamphaeng Phet | | | Dengue virus |  | | | | |  |  | | | 23/1000 (2.3) | Blood/IgM/IgG ELISA (in-house), RT-PCR, Culture | | | | | | |  |
| **VIET NAM** | | |  | |  | |  | | | | | |  |  | | | | | | |  |
| Kien Giang | | | *S*. *enterica* serovar Typhi | | 0-59 y | | | | Outpatient | Suspected enteric fever | | | 207/425 (48.7) | Blood/Stool/Culture | | | | | | Tran T. *et al.* 1995 | |
|  | | | *S*. *enterica* serovar Paratyphi A | |  | | | |  |  | | | 19/425 (4.5) | Blood/Stool/Culture | | | | | |  | |
|  | | | *Salmonella choleraesuis* | |  | | | |  |  | | | 2/425 (0.5) | Blood/Stool/Culture | | | | | |  | |
| Ho Chi Minh | | | *S. enterica* serovar *Typhi* | | 1-15 y | | | | Inpatient | Suspected typhoid fever | | | 100/108 (92.6) | Blood/Urine/Stool/Culture | | | | | | Vinh H. *et al.* 1996 | |
| Ha Nam | | *Salmonella* ssp | | ≥15 y | | | | Inpatient | Suspected enteric fever | | | 77/171 (45.0) | Blood/Urine/Culture, MAb-based dot-blot ELISA, Widal agglutination test | | | | | | Nguyen N. *et al.* 1997 | |  |
| Dong Nai | | Dengue virus | | 5-8 y | | | | Inpatient | Fever or history of fever | | | 443/548 (80.8) | Blood/IgM ELISA (in-house) | | | | | | Bethell D. *et al.* 1998 | |  |
| Ho Chi Minh | | *S*. *enterica* serovar Typhi | | All ages | | | | Inpatient | N/A | | | 294/437 (67.3) | Blood/Culture | | | | | | Hoa N. *et al.* 1998 | |  |
|  | | *Escherichia coli* | |  | | | |  |  | | | 45/437 (10.3) | Blood/Culture | | | | | |  | |  |
|  | | *Staphylococcus aureus* | |  | | | |  |  | | | 24/437 (5.5) | Blood/Culture | | | | | |  | |  |
|  | | *Klebsiella* spp | |  | | | |  |  | | | 21/437 (4.8) | Blood/Culture | | | | | |  | |  |
|  | | *S*. *enterica* serovar Paratyphi A | |  | | | |  |  | | | 15/437 (3.4) | Blood/Culture | | | | | |  | |  |
|  | | Streptococci | |  | | | |  |  | | | 13/437 (3.0) | Blood/Culture | | | | | |  | |  |
|  | | *Staphylococcus pneumoniae* | |  | | | |  |  | | | 8/437 (1.8) | Blood/Culture | | | | | |  | |  |
|  | | *Pseudomonas aeruginosa* | |  | | | |  |  | | | 4/437 (0.9) | Blood/Culture | | | | | |  | |  |
|  | | *Salmonella choleraesuis* | |  | | | |  |  | | | 4/437 (0.9) | Blood/Culture | | | | | |  | |  |
|  | | *Aeromonas hydrophila* | |  | | | |  |  | | | 2/437 (0.5) | Blood/Culture | | | | | |  | |  |
|  | | *Neisseria meningitides* | |  | | | |  |  | | | 2/437 (0.5) | Blood/Culture | | | | | |  | |  |
|  | | *Salmonella* ssp | |  | | | |  |  | | | 2/437 (0.5) | Blood/Culture | | | | | |  | |  |
|  | | *Cryptococcus neoformans* | |  | | | |  |  | | | 1/437 (0.2) | Blood/Culture | | | | | |  | |  |
|  | | *Haemophilus influenza* | |  | | | |  |  | | | 1/437 (0.2) | Blood/Culture | | | | | |  | |  |
|  | | *Proteus mirabilis* | |  | | | |  |  | | | 1/437 (0.2) | Blood/Culture | | | | | |  | |  |
| Ho Chi Minh | | Japanese encephalitis virus | | All ages | | | | Inpatient | Suspected encephalitis | | | 60/181 (33.2) | Blood/JE MAC DOT/JE MAC ELISA | | | | | | Solomon T. *et al.* 1998 | |  |
|  | | Dengue virus | |  | | | |  |  | | | 3/181 (1.7) | Blood/IgM ELISA (Venture and Innis) | | | | | |  | |  |
| Tien Giang | | *Leptospira* | | 15-60 y | | | | Population survey (asymptomatic)^⧫^ | N/A | | | 41/1400 (2.9) | Blood/MAT | | | | | | Van C. *et al.* 1998 | |  |
| Ho Chi Minh | | *Burkholderia pseudomallei* | | All ages | | | | Inpatient | N/A | | | 9/3653 (0.3) | Blood/Culture | | | | | | Parry C. *et al.* 1999 | |  |
| Ho Chi Minh | | *Plasmodium falciparum* | | 1-90 y | | | | Inpatient | N/A | | | 103/290 (35.5) | Blood/Blood smear Giemsa microscopy | | | | | | Parry C. *et al.* 1999 | |  |
|  | | *S*. *enterica* serovar Typhi | |  | | | |  |  | | | 154/500 (30.8) | Blood/Widal agglutination test, Culture | | | | | |  | |  |
|  | | Dengue virus | |  | | | |  |  | | | 76/290 (26.2) | Blood/IgM/IgG rapid test (PanBio) | | | | | |  | |  |
|  | | *Escherichia coli* | |  | | | |  |  | | | 45/290 (15.5) | Blood/Culture | | | | | |  | |  |
|  | | *S*. *enterica* serovar Paratyphi A | |  | | | |  |  | | | 45/500 (9.0) | Blood/Widal agglutination test, Culture | | | | | |  | |  |
|  | | Streptococci | |  | | | |  |  | | | 21/290 (7.2) | Blood/Culture | | | | | |  | |  |
|  | | *Staphylococcus aureus* | |  | | | |  |  | | | 15/290 (5.2) | Blood/Culture | | | | | |  | |  |
|  | | *Salmonella* ssp | |  | | | |  |  | | | 10/290 (3.5) | Blood/Culture | | | | | |  | |  |
|  | | *Klebsiella* spp | |  | | | |  |  | | | 9/290 (3.1) | Blood/Culture | | | | | |  | |  |
|  | | *Pseudomonas aeruginosa* | |  | | | |  |  | | | 3/290 (1.0) | Blood/Culture | | | | | |  | |  |
|  | | *Acinetobacter* spp | |  | | | |  |  | | | 2/290 (0.7) | Blood/Culture | | | | | |  | |  |
|  | | *Cryptococcus neoformans* | |  | | | |  |  | | | 2/290 (0.7) | Blood/Culture | | | | | |  | |  |
|  | | *Aeromonas hydrophila* | |  | | | |  |  | | | 1/290 (0.3) | Blood/Culture | | | | | |  | |  |
|  | | *Burkholderia pseudomallei* | |  | | | |  |  | | | 1/290 (0.3) | Blood/Culture | | | | | |  | |  |
|  | | *Penicillium marneffei* | |  | | | |  |  | | | 1/290 (0.3) | Blood/Culture | | | | | |  | |  |
|  | | *Proteus mirabilis* | |  | | | |  |  | | | 1/290 (0.3) | Blood/Culture | | | | | |  | |  |
| Ho Chi Minh | | *S*. *enterica* serovar Typhi | | ≥15 y | | | | Inpatient | Suspected enteric fever | | | 86/97 (88.7) | Blood/Culture | | | | | | Chinh N. *et al.* 2000 | |  |
|  | | *S*. *enterica* serovar Paratyphi | |  | | | |  |  | | | 2/97 (2.1) | Blood/Culture | | | | | |  | |  |
| Dong Thap | | *S*. *enterica* serovar Typhi | | 0.25-87 y | | | | In-and outpatient | ≥3 days of fever (≥38.5°C) | | | 56/658 (8.5) | Blood/Culture | | | | | | Lin F. *et al.* 2000 | |  |
| Dong Thap | | *S*. *enterica* serovar Typhi | | 4-42 y | | | | Inpatient | >4 days of fever | | | 89/120 (74.2) | Blood/Bone-marrow/Culture | | | | | | Wain J. *et al.* 2001 | |  |
|  | | *S*. *enterica* serovar Paratyphi A | |  | | | |  |  | | | 1/120 (0.8) | Blood/Bone-marrow/Culture | | | | | |  | |  |
| Ho Chi Minh | | Dengue virus | | <1 y | | | | Inpatient | Suspected dengue infection | | | 652/14053 (4.6) | Blood/IgG/IgM ELISA (AFRIMS), haemagglutination inhibition assay | | | | | | Halstead S. *et al.* 2002 | |  |
| Hanoi | | *Leptospira* | | All ages | | | | Not specified | N/A | | | 23/288 (8.0) | Blood/MAT, PCR | | | | | | Laras K. *et al.* 2002 | |  |
| Ho Chi Minh | | *Leptospira* | |  | | | |  |  | | | 4/266 (1.5) | Blood/MAT, PCR | | | | | |  | |  |
| An Giang | | *Leptospira* | |  | | | |  |  | | | 7/464 (1.5) | Blood/MAT, PCR | | | | | |  | |  |
| Ho Chi Minh | | Japanese encephalitis virus | | All ages | | | | Inpatient | Suspected CNS infections | | | 144/555 (26.0) | Blood/CSF/JEV IgM ELISA, Culture | | | | | | Solomon T. *et al.* 2002 | |  |
| Dong Nai | | Dengue virus | | 3-13 y | | | | Inpatient | <7 days of fever | | | 712/1136 (62.7) | Blood/IgM/IgG ELISA (AFRIMS), RT-PCR, viral isolation | | | | | | Phuong C. *et al.* 2004 | |  |
|  | | *S*. *enterica* serovar Typhi | |  | | | |  |  | | | 6/85 (7.1) | Blood/Culture | | | | | |  | |  |
|  | | *Plasmodium falciparum* | |  | | | |  |  | | | 1/85 (1.2) | Blood/Blood smear Giemsa microscopy | | | | | |  | |  |
| Binh Thuan | | Dengue virus | | 8-15 y | | | | Inpatient | Suspected dengue infection | | | 40/50 (80.0) | Blood/IgM/IgG ELISA (in-house), haemagglutination inhibition assay | | | | | | Buchy P. *et al.* 2005 | |  |
|  | | Dengue virus | |  | | | |  |  | | | 11/50 (22.0) | Blood/Viral isolation, RT-PCR | | | | | |  | |  |
| Khanh Hoa | | Dengue virus | |  | | | |  |  | | | 68/75 (90.7) | Blood/IgM/IgG ELISA (in-house), haemagglutination inhibition assay | | | | | |  | |  |
|  | | Dengue virus | |  | | | |  |  | | | 15/75 (20.0) | Blood/Viral isolation, RT-PCR | | | | | |  | |  |
| Ho Chi Minh | | Dengue virus | | <1 y | | | | Inpatient | Suspected dengue infection | | | 245/272 (90.1) | Blood/IgM/IgG ELISA (in-house) | | | | | | Nguyen T. *et al.* 2005 | |  |
| Son La | | *S*. *enterica* serovar Typhi | | 0-79 y | | | | Not specified | >3 days of fever (>38°C), negative malaria smear | | | 90/617 (14.6) | Blood/Culture | | | | | | Tran H. *et al.* 2005 | |  |
| Binh Thuan | | Dengue virus | | 4-82 y | | | | Outpatient | >14 days of fever (≥38°C) | | | 234/697 (33.6) | Blood/IgM/IgG ELISA (Focus) | | | | | | Phuong H. *et al.* 2006 | |  |
| Binh Thuan | | Dengue virus | | All ages | | | | Outpatient | >14 days of fever (≥38°C) | | | 272/781 (34.8) | Blood/IgM/IgG ELISA (Focus) | | | | | | Tran T. *et al.* 2006 | |  |
| Binh Thuan | | Dengue virus | | 7-14 y | | | | Population survey (asymptomatic)^⧫^ | N/A | | | 66/216 (30.6) | Blood/IgG ELISA (Focus), IgG IFA (PanBio) | | | | | | Thai K. *et al.* 2007 | |  |
| Ho Chi Minh, Dong Thap, An Giang | | *S*. *enterica* serovar Typhi | | 1-42 y | | | | In-and outpatient | Suspected typhoid fever | | | 287/358 (80.2) | Blood/Culture | | | | | | Dolecek C. *et al.* 2008 | |  |
| Ho Chi Minh | | Japanese encephalitis virus | | 0.2-85 y | | | | Inpatient | Suspected CNS infection | | | 63/380 (16.6) | Blood/CSF/JE IgM/IgG ELISA, Culture, PCR | | | | | | Solomon T. *et al.* 2008 | |  |
| Ho Chi Minh | | Dengue virus | | 4-42 y | | | | Inpatient | Suspected dengue infection | | | 125/138 (90.6) | Blood/IgM/IgG ELISA (Venture), NS1 antigen (BioRad), NS1 rapid test (BioRad), qRT-PCR | | | | | | Hang V. *et al.* 2009 | |  |
| Thua Thien-Hue | | *Orientia tsutsugamushi* | | All ages | | | | Inpatient | Suspected scrub typhus | | | 41/48 (85.4) | Blood/IFA, RT-PCR | | | | | | Kramme S. *et al.* 2009 | |  |
| Ho Chi Minh | | Dengue virus | | <1.5 y | | | | Inpatient | Suspected dengue infection | | | 161/201 (80.1) | Blood/qRT-PCR | | | | | | Chau T. *et al.* 2010 | |  |
|  | | Dengue virus | |  | | | |  |  | | | 204/303 (67.3) | Blood/IgM/IgG ELISA(Venture), NS1 antigen(BioRad), plaque reduction neutralization assay | | | | | |  | |  |
| Ho Chi Minh | | Japanese encephalitis virus | | <16 y | | | | Inpatient | Acute encephalitis | | | 50/194 (25.8) | CSF/JE capture IgM ELISA, Culture, PCR | | | | | | Le V. *et al.* 2010 | |  |
|  | | Enterovirus | |  | | | |  |  | | | 4/194 (2.1) | CSF/Blood/Rectal/ Throat/PCR | | | | | |  | |  |
|  | | Dengue virus | |  | | | |  |  | | | 7/194 (3.6) | CSF/IgM ELISA (Venture) | | | | | |  | |  |
|  | | Dengue virus | |  | | | |  |  | | | 6/194 (3.1) | CSF/Viral isolation | | | | | |  | |  |
|  | | Dengue virus | |  | | | |  |  | | | 4/194 (2.1) | CSF/PCR | | | | | |  | |  |
|  | | *H. influenzae* type b | |  | | | |  |  | | | 6/163 (3.7) | CSF/PCR | | | | | |  | |  |
|  | | *Streptococcus pneumoniae* | |  | | | |  |  | | | 6/163 (3.7) | CSF/PCR | | | | | |  | |  |
|  | | Cytomegalo-virus | |  | | | |  |  | | | 1/194 (0.5) | CSF/Culture, PCR/RT-PCR | | | | | |  | |  |
|  | | Herpes simplex virus | |  | | | |  |  | | | 1/194 (0.5) | CSF/Culture, PCR/RT-PCR | | | | | |  | |  |
|  | | Influenza A virus | |  | | | |  |  | | | 1/194 (0.5) | CSF/Culture, PCR/RT-PCR | | | | | |  | |  |
| Binh Thuan | | Dengue virus | | 1-85 y | | | | Population survey (symptomatic) | <14 days of fever (≥38°C) | | | 176/1067 (16.5) | Blood/IgM/IgG ELISA (Focus), IgM/IgG rapid test (PanBio) | | | | | | Phuong H. *et al.* 2010 | |  |
| Binh Thuan | | Dengue virus | | 0.1-95.1 y | | | | Population survey (symptomatic) | Fever | | | 86/351 (24.5) | Blood/qRT-PCR | | | | | | Thai K. *et al.* 2010 | |  |
|  | | Dengue virus | |  | | | |  |  | | | 351/1938 (18.1) | Blood/IgM/IgG ELISA (Focus), NS1 antigen (BioRad) | | | | | |  | |  |
| An Giang | | Dengue virus | | 2-15 y | | | | Population survey (symptomatic) | Fever | | | 310/627 (49.4) | Blood/IgM/IgG ELISA (in-house), NS1 antigen (BioRad), viral isolation, qRT-PCR | | | | | | Tien N. *et al.* 2010 | |  |
| Ho Chi Minh | | Dengue virus | | >14 y | | | | Inpatient | Suspected dengue infection | | | 644/740 (87.0) | Blood/IgM/IgG ELISA (Venture), NS1 antigen (BioRad), RT-PCR | | | | | | Trung D. *et al.* 2010 | |  |
| Ho Chi Minh | | Dengue virus: | | ≤15 y | | | | Inpatient | Suspected dengue infection | | |  | Blood/IgM ELISA (Inverness) (Venture), RT-PCR | | | | | | Anders K. *et al.* 2011 | |  |
|  | | (2004) | |  | | | |  |  | | | 306/419 (73.0) |  | | | | | |  | |  |
|  | | (2005) | |  | | | |  |  | | | 494/655 (75.4) |  | | | | | |  | |  |
|  | | (2006) | |  | | | |  |  | | | 983/1540 (63.8) |  | | | | | |  | |  |
|  | | (2007) | |  | | | |  |  | | | 2002/2769 (72.3) |  | | | | | |  | |  |
|  | | (2008) | |  | | | |  |  | | | 3029/4012 (75.5) |  | | | | | |  | |  |
|  | | (2009) | |  | | | |  |  | | | 1604/2342 (68.5) |  | | | | | |  | |  |
| Ho Chi Minh | | Dengue virus | | 5-15 y | | | | Inpatient | <3 days of fever | | | 203/301 (67.4) | Blood/RT-PCR, qRT-PCR | | | | | | Duyen HT. *et al.* 2011 | |  |
|  | |  | |  | | | |  |  | | | 8/301 (2.7) | Blood/IgM/IgG ELISA (Venture), NS1 antigen (BioRad) | | | | | |  | |  |
| Hanoi | | Dengue virus | | 18-31 y | | | | Inpatient | Fever plus two or more of the following: headache; retro-orbital pain; myalgia/arthralgia; rash; bleeding or leukopenia | | | 123/158 (77.9) | Blood/IgM/G capture ELISA (Venture), NS1 antigen (BioRad) | | | | | | Fox A. *et al.* 2011 | |  |
|  | | Dengue virus | |  | | | |  |  | | | 81/130 (62.3) | Blood/qRT-PCR | | | | | |  | |  |
| **LAO PDR** | | | | | | | | | | | | | | | | | | | | |  |
| Vientiane | | *Leptospira* | | 3-73 y | | | | In-and outpatient | Acute jaundice | 15/70 (21.4) | | | Blood/*Leptospira* IgM/IgG ELISA | | | | | Bounlu K. *et al.* 1998 | | |  |
|  | | Hepatitis virus A | |  | | | |  |  | 26/186 (14.0) | | | Blood/Anti-HAV IgM ELISA | | | | |  | | |  |
|  | | Hepatitis E | |  | | | |  |  | 21/186 (11.3) | | | Blood/Anti-HEV IgG ELISA, IgM ELISA, PCR | | | | |  | | |  |
|  | | Hepatitis virus B | |  | | | |  |  | 19/186 (10.2) | | | Blood/Anti-HBsAg IgM ELISA | | | | |  | | |  |
|  | | Hepatitis C | |  | | | |  |  | 14/186 (7.5) | | | Blood/Anti-HCV IgG ELISA | | | | |  | | |  |
|  | | Hepatitis G | |  | | | |  |  | 2/88 (2.3) | | | Blood/RT-PCR | | | | |  | | |  |
| Vientiane | | *Leptospira* | | All ages | | | | Not specified | N/A | 33/232 (14.2) | | | Blood/MAT, PCR | | | | | Laras K. *et al.* 2002 | | |  |
| Vientiane | | *Leptospira* | | All ages | | | | Inpatient | N/A | 23/186 (12.4) | | | Blood/MAT | | | | | Blacksell S. *et al.* 2006 | | |  |
| Vientiane | | *S*. *enterica* serovar Typhi | | All ages | | | | Inpatient | Suspected community-acquired bacteremia | 246/4460 (5.5) | | | Blood/Culture | | | | | Phetsouvanh R. *et al.* 2006 | | |  |
|  | | *Staphylococcus aureus* | |  | | | |  |  | 92/4460 (2.1) | | | Blood/Culture | | | | |  | | |  |
|  | | *Escherichia coli* | |  | | | |  |  | 60/4460 (1.4) | | | Blood/Culture | | | | |  | | |  |
|  | | *Klebsiella pneumoniae* | |  | | | |  |  | 20/4460 (0.5) | | | Blood/Culture | | | | |  | | |  |
|  | | *Burkholderia pseudomallei* | |  | | | |  |  | 14/4460 (0.3) | | | Blood/Culture | | | | |  | | |  |
|  | | *Pseudomonas aeruginosa* | |  | | | |  |  | 13/4460 (0.3) | | | Blood/Culture | | | | |  | | |  |
|  | | *Salmonella* spp | |  | | | |  |  | 9/4460 (0.2) | | | Blood/Culture | | | | |  | | |  |
|  | | *Aeromonas hydrophila* | |  | | | |  |  | 3/4460 (0.1) | | | Blood/Culture | | | | |  | | |  |
|  | | *Salmonella choleraesuis* | |  | | | |  |  | 3/4460 (0.1) | | | Blood/Culture | | | | |  | | |  |
|  | | *Streptococcus* Group C | |  | | | |  |  | 3/4460 (0.1) | | | Blood/Culture | | | | |  | | |  |
|  | | *Streptococcus pneumoniae* | |  | | | |  |  | 6/4460 (0.1) | | | Blood/Culture | | | | |  | | |  |
|  | | *Streptococcus* spp | |  | | | |  |  | 2/4460 (0.04) | | | Blood/Culture | | | | |  | | |  |
|  | | Unspeciated viridans streptococci | |  | | | |  |  | 2/4460 (0.04) | | | Blood/Culture | | | | |  | | |  |
|  | | *Candida* spp | |  | | | |  |  | 1/4460 (0.02) | | | Blood/Culture | | | | |  | | |  |
|  | | *Cryptococcus neoformans* | |  | | | |  |  | 1/4460 (0.02) | | | Blood/Culture | | | | |  | | |  |
|  | | *Citrobacter freundii* | |  | | | |  |  | 1/4460 (0.02) | | | Blood/Culture | | | | |  | | |  |
|  | | *Edwardsiella tarda* | |  | | | |  |  | 1/4460 (0.02) | | | Blood/Culture | | | | |  | | |  |
|  | | *Proteus vulgaris* | |  | | | |  |  | 1/4460 (0.02) | | | Blood/Culture | | | | |  | | |  |
|  | | *Haemophilus influenza* | |  | | | |  |  | 1/4460 (0.02) | | | Blood/Culture | | | | |  | | |  |
|  | | *Shigella flexneri* | |  | | | |  |  | 1/4460 (0.02) | | | Blood/Culture | | | | |  | | |  |
|  | | *Streptococcus bovis* | |  | | | |  |  | 1/4460 (0.02) | | | Blood/Culture | | | | |  | | |  |
|  | | *Streptococcus* Group B | |  | | | |  |  | 1/4460 (0.02) | | | Blood/Culture | | | | |  | | |  |
|  | | *Streptococcus* Group G | |  | | | |  |  | 1/4460 (0.02) | | | Blood/Culture | | | | |  | | |  |
|  | | *Streptococcus milleri* | |  | | | |  |  | 1/4460 (0.02) | | | Blood/Culture | | | | |  | | |  |
|  | | *Acinetobacter* spp | |  | | | |  |  | 0/4460 (0.0) | | | Blood/Culture | | | | |  | | |  |
|  | | *Aerococcus viridians* | |  | | | |  |  | 0/4460 (0.0) | | | Blood/Culture | | | | |  | | |  |
|  | | *Burkholderia cepacia* | |  | | | |  |  | 0/4460 (0.0) | | | Blood/Culture | | | | |  | | |  |
|  | | Coagulase negative *Staphylococcus* | |  | | | |  |  | 0/4460 (0.0) | | | Blood/Culture | | | | |  | | |  |
|  | | *Enterobacter* spp | |  | | | |  |  | 0/4460 (0.0) | | | Blood/Culture | | | | |  | | |  |
|  | | *Enterococcus* spp | |  | | | |  |  | 0/4460 (0.0) | | | Blood/Culture | | | | |  | | |  |
|  | | *Klebsiella* spp | |  | | | |  |  | 0/4460 (0.0) | | | Blood/Culture | | | | |  | | |  |
|  | | *Ochrobactrum anthropi* | |  | | | |  |  | 0/4460 (0.0) | | | Blood/Culture | | | | |  | | |  |
|  | | *Pseudomonas* spp | |  | | | |  |  | 0/4460 (0.0) | | | Blood/Culture | | | | |  | | |  |
| Vientiane | | *Orientia tsutsugamushi* | | >15 y | | | | Inpatient | Fever | 63/427 (14.8) | | | Blood/IFA | | | | | Phongmany S. *et al.* 2006 | | |  |
|  | | *Rickettsia typhi* | |  | | | |  |  | 41/427 (9.6) | | | Blood/IFA | | | | |  | | |  |
|  | | *Rickettsia helvetica* | |  | | | |  |  | 8/427 (1.9) | | | Blood/IFA | | | | |  | | |  |
|  | | *Rickettsia* 'AT1' from Japan | |  | | | |  |  | 1/427 (0.2) | | | Blood/IFA | | | | |  | | |  |
|  | | *Rickettsia conorii* | |  | | | |  |  | 1/427 (0.2) | | | Blood/IFA | | | | |  | | |  |
|  | | *Rickettsia felis* | |  | | | |  |  | 1/427 (0.2) | | | Blood/IFA | | | | |  | | |  |
|  | | *Rickettsia heilongjiangensis* | |  | | | |  |  | 0/127 (0.0) | | | Blood/IFA | | | | |  | | |  |
|  | | *Rickettsia honei* | |  | | | |  |  | 0/127 (0.0) | | | Blood/IFA | | | | |  | | |  |
|  | | *Rickettsia japonica* | |  | | | |  |  | 0/127 (0.0) | | | Blood/IFA | | | | |  | | |  |
|  | | *Rickettsia slovaca* | |  | | | |  |  | 0/127 (0.0) | | | Blood/IFA | | | | |  | | |  |
| Vientiane | | Dengue virus | | All ages | | | | Inpatient | Suspected dengue infection | 25/38 (65.8) | | | Blood/RT-PCR | | | | | Blacksell S. *et al.* 2007 | | |  |
|  | | Dengue virus | |  | | | |  |  | 38/87 (43.7) | | | Blood/IgM/IgG ELISA (AFRIMS), 8 different rapid tests | | | | |  | | |  |
|  | | *Orientia tsutsugamushi* | |  | | | |  |  | 12/87 (13.8) | | | Blood/IFA | | | | |  | | |  |
|  | | *Rickettsia typhi* | |  | | | |  |  | 4/87 (4.6) | | | Blood/IFA | | | | |  | | |  |
|  | | Japanese encephalitis virus | |  | | | |  |  | 1/87 (1.2) | | | Blood/JE IgM/IgG ELISA (AFRIMS) | | | | |  | | |  |
|  | | Chikungunya virus | |  | | | |  |  | 0/87 (0.0) | | | Blood/ Haemagglutination inhibition assay | | | | |  | | |  |
| Vientiane | | *Rickettsia typhi* | | >15 y | | | | Inpatient | Negative malaria smear, fever ≥37.5°C | 23/53 (43.4) | | | Blood/IFA | | | | | Phetsouvanh R. *et al.* 2009 | | |  |
|  | | *Orientia tsutsugamushi* | |  | | | |  |  | 15/53 (28.3) | | | Blood/IFA | | | | |  | | |  |
|  | | *S*. *enterica* serovar Typhi | |  | | | |  |  | 7/53 (13.2) | | | Blood/Culture | | | | |  | | |  |
| Vientiane | | Dengue virus | | Child (0.5 – 6 yrs), adult (≥35 yrs) | | | | Population survey (asymptomatic)^⧫^ | N/A | 126/3558 (3.5%) | | | Blood/IgM/IgG ELISA (in-house) | | | | | Vallée J. *et al.* 2009 | | |  |
|  | | Japanese encephalitis virus | |  | | | |  |  | 78/3558 (2.2%) | | | Blood/IgM/IgG ELISA (in-house) | | | | |  | | |  |
| Vientiane | | *Orientia tsutsugamushi* | | All ages | | | | Inpatient | Fever, headache and/or myalgia | 426/1030 (41.4) | | | Blood/IFA | | | | | Blacksell S *et al.* 2010 | | |  |
|  | | *Rickettsia typhi* | |  | | | |  |  | 409/1030 (39.7) | | | Blood/IFA | | | | |  | | |  |
| Vientiane | | Hepatitis virus A | | All ages | | | | Inpatient | Acute jaundice or elevated AST^3^ or ALT^4^ | 115/300 (38.3) | | | Blood/Vidas Immunoassay/Anti-HAV IgM | | | | | Syhavong B. *et al.* 2010 | | |  |
|  | | Dengue virus | |  | | | |  |  | 30/139 (21.6) | | | Blood/IgM/IgG ELISA (PanBio), NS1 antigen (PanBio) | | | | |  | | |  |
|  | | Hepatitis virus C | |  | | | |  |  | 46/304 (15.1) | | | Blood/Vidas Immunoassay/Anti-HCV IgM, PCR | | | | |  | | |  |
|  | | Hepatitis virus B | |  | | | |  |  | 11/93 (11.8) | | | Blood/Vidas Immunoassay/Anti-HBsAg/IgM | | | | |  | | |  |
|  | | *Leptospira* | |  | | | |  |  | 24/303 (7.9) | | | Blood/MAT | | | | |  | | |  |
|  | | *Rickettsia typhi* | |  | | | |  |  | 11/301 (3.7) | | | Blood/IFA | | | | |  | | |  |
|  | | *Escherichia coli* | |  | | | |  |  | 5/166 (3.0) | | | Blood/Culture | | | | |  | | |  |
|  | | *S*. *enterica* serovar Typhi | |  | | | |  |  | 5/166 (3.0) | | | Blood/Culture | | | | |  | | |  |
|  | | *Orientia tsutsugamushi* | |  | | | |  |  | 8/301 (2.7) | | | Blood/IFA | | | | |  | | |  |
|  | | Hepatitis virus E | |  | | | |  |  | 5/298 (1.7) | | | Blood/HEV IgM/Total Ig AFRIMS ELISA, PCR | | | | |  | | |  |
|  | | Spotted fever group rickettsia | |  | | | |  |  | 5/301 (1.7) | | | Blood/IFA | | | | |  | | |  |
|  | | *Staphylococcus aureus* | |  | | | |  |  | 2/166 (1.2) | | | Blood/Culture | | | | |  | | |  |
|  | | *Burkholderia pseudomallei* | |  | | | |  |  | 1/166 (0.6) | | | Blood/Culture | | | | |  | | |  |
|  | | *Plasmodium falciparum* | |  | | | |  |  | 0/68 (0.0) | | | Blood/Blood smear Giemsa microscopy | | | | |  | | |  |
| Vientiane | | Dengue virus | | >15 y | | | | Inpatient | Fever of<7 days | 170/234 (72.7) | | | Blood/IgM/IgG ELISA (PanBio), NS1 antigen (PanBio) | | | | | Mayxay M. *et al.* 2011 | | |  |
|  | | *Orientia tsutsugamushi* | |  | | | |  |  | 25/234 (10.7) | | | Blood/IFA | | | | |  | | |  |
|  | | *Rickettsia typhi* | |  | | | |  |  | 7/234 (3.0) | | | Blood/IFA | | | | |  | | |  |
|  | | Japanese encephalitis virus | |  | | | |  |  | 3/234 (1.3) | | | Blood/PanBio JE/Dengue IgM Combo ELISA | | | | |  | | |  |
|  | | *S*. *enterica* serovar Typhi | |  | | | |  |  | 2/234 (0.9) | | | Blood/Culture | | | | |  | | |  |
| **CAMBODIA** | | | | | | | | | | | | | | | | | | | | |  |
| Phnom Penh | | Dengue virus | | | 2-15 y | | Inpatient | | N/A | | 22/39 (56.4) | | | Blood/IgM/IgG ELISA (Innis), viral isolation, RT-PCR | | | Rathavuth H. *et al.* 1997 | | | |  |
| Kandal | | Dengue virus | | |  | |  | |  | | 9/39 (23.1) | | |  | | |  | | | |  |
| Takeo | | Dengue virus | | |  | |  | |  | | 2/39 (5.1) | | |  | | |  | | | |  |
| Kampong Speu | | Dengue virus | | |  | |  | |  | | 2/39 (5.1) | | |  | | |  | | | |  |
| Kampong Cham | | Dengue virus | | |  | |  | |  | | 1/39 (2.6) | | |  | | |  | | | |  |
| Kampot | | Dengue virus | | |  | |  | |  | | 1/39 (2.6) | | |  | | |  | | | |  |
| Kampong Thom | | Dengue virus | | |  | |  | |  | | 1/39 (2.6) | | |  | | |  | | | |  |
| Prey Veng | | Dengue virus | | |  | |  | |  | | 1/39 (2.6) | | |  | | |  | | | |  |
| Phnom Penh, Kandal, Takeo, Kampong Speu, Kampong Cham, Prey Veng | | Dengue virus | | | ≤14 y | | Inpatient | | Signs of hemorrhagic fever or encephalitis or hepatitis | | 509/621 (82.0) | | | Blood/IgM/IgG ELISA (Innis), viral isolation | | | Chhour Y. *et al.* 2002 | | | |  |
|  | | Dengue virus | | |  | |  | |  | | 22/75 (29.3) | | | Blood/PCR | | |  | | | |  |
|  | | Hepatitis virus A | | |  | |  | |  | | 24/44 (54.6) | | | Blood/Anti-HAV IgM ELISA | | |  | | | |  |
|  | | Japanese encephalitis virus | | |  | |  | |  | | 9/50 (18.0) | | | Blood/JE IgM/IgG ELISA, Culture | | |  | | | |  |
|  | | Hepatitis virus B | | |  | |  | |  | | 1/44 (2.3) | | | Blood/Anti-HBsAg IgM ELISA | | |  | | | |  |
|  | | Hepatitis virus C | | |  | |  | |  | | 0/44 (0.0) | | | Blood/ELISA total anti-HCV Ig | | |  | | | |  |
|  | | Hepatitis virus E | | |  | |  | |  | | 0/44 (0.0) | | | Blood/ELISA total Ig and IgM to HEV | | |  | | | |  |
| Phnom Penh | | *Leptospira* | | | All ages | | Not specified | | N/A | | 24/202 (11.9) | | | Blood/*Leptospira* IgM ELISA | | | Laras K. *et al.* 2002 | | | |  |
|  | | *Leptospira* | | |  | |  | |  | | 6/202 (3%) | | | Blood/MAT, PCR | | |  | | | |  |
| Takeo | | Adults: | | | >15 y | | Inpatient | | Fever or history of fever and clinical encephalitis syndrome | |  | | |  | | | Srey V. *et al.* 2002 | | | |  |
|  | | *Cryptococcus neoformans* | | |  | |  | |  | | 7/47 (14.9) | | | Blood/CSF/Culture | | |  | | | |  |
|  | | *Mycobacterium tuberculosis* | | |  | |  | |  | | 4/47 (8.5) | | | Blood/CSF/Culture | | |  | | | |  |
|  | | *Streptococcus* spp | | |  | |  | |  | | 2/47 (4.3) | | | Blood/CSF/Culture | | |  | | | |  |
|  | | Dengue virus | | |  | |  | |  | | 1/47 (2.1) | | | Blood/CSF/IgM ELISA (in-house), haemagglutination inhibition assay, viral isolation, RT-PCR | | |  | | | |  |
|  | | Herpes simplex virus | | |  | |  | |  | | 2/47 (4.3) | | | Blood/CSF/PCR | | |  | | | |  |
|  | | Flavivirus and Alphavirus | | |  | |  | |  | | 2/47 (4.3) | | | Blood/CSF/Culture | | |  | | | |  |
|  | | Children: | | | <15 y | |  | |  | |  | | |  | | |  | | | |  |
|  | | *Haemophilus influenza* | | |  | |  | |  | | 3/52 (5.8) | | | Blood/CSF/Culture | | |  | | | |  |
|  | | *Staphylococcus* spp | | |  | |  | |  | | 1/52 (1.9) | | | Blood/CSF/Culture | | |  | | | |  |
|  | | *Mycobacterium tuberculosis* | | |  | |  | |  | | 1/52 (1.9) | | | Blood/CSF/Culture | | |  | | | |  |
|  | | Dengue virus | | |  | |  | |  | | 4/52 (2.1) | | | Blood/CSF/IgM ELISA (in-house), haemagglutination inhibition assay, viral isolation, RT-PCR | | |  | | | |  |
|  | | Japanese encephalitis virus | | |  | |  | |  | | 16/52 (30.8) | | | Blood/CSF/IgM ELISA (in-house), haemagglutination inhibition assay | | |  | | | |  |
|  | | Japanese encephalitis virus | | |  | |  | |  | | 0/16 (0) | | | Blood/CSF/Viral isolation, RT-PCR | | |  | | | |  |
| Takeo | | *Leptospira* | | | 16-60 y | | Not specified | | Fever or headache or myalgia | | 11/121 (9.1) | | | Blood/MAT, Culture, PCR | | | Seng H *et al.* 2007 | | | |  |
| Svay Rieng | | Japanese encephalitis virus | | | ≤15 y | | Inpatient | | Syndromic meningoencephalitis | | 13/37 (35.1) | | | Blood/CSF/JE–Dengue IgM Combo ELISA (Panbio Diagnostics) | | | Touch S. *et al.* 2009 | | | |  |
| Takeo | |  | | |  | |  | |  | | 16/75 (21.3) | | |  | | |  | | | |  |
| Siem Reap | |  | | |  | |  | |  | | 43/215 (20.0) | | |  | | |  | | | |  |
| Phnom Penh | |  | | |  | |  | |  | | 21/139 (15.1) | | |  | | |  | | | |  |
| Kampong Cham | |  | | |  | |  | |  | | 13/89 (14.6) | | |  | | |  | | | |  |
| Battambang | |  | | |  | |  | |  | | 4/31 (12.9) | | |  | | |  | | | |  |
| Siem Reap | | Dengue virus | | | 0-19 y | | Population survey (symptomatic) | | N/A | |  | | | Blood/IgM ELISA (in-house), haemagglutination inhibition assay, viral isolation, RT-PCR | | | Huy R. *et al.* 2010 | | | |  |
|  | | (2000) | | |  | |  | |  | | 324/415 (78.1) | | |  | | |  | | | |  |
|  | | (2001) | | |  | |  | |  | | 603/748 (80.6) | | |  | | |  | | | |  |
|  | | (2002) | | |  | |  | |  | | 736/809 (91.0) | | |  | | |  | | | |  |
|  | | (2003) | | |  | |  | |  | | 617/677 (91.1) | | |  | | |  | | | |  |
|  | | (2004) | | |  | |  | |  | | 611/680 (89.9) | | |  | | |  | | | |  |
|  | | (2005) | | |  | |  | |  | | 467/527 (88.6) | | |  | | |  | | | |  |
|  | | (2006) | | |  | |  | |  | | 510/575 (88.7) | | |  | | |  | | | |  |
|  | | (2007) | | |  | |  | |  | | 1315/1400 (93.9) | | |  | | |  | | | |  |
|  | | (2008) | | |  | |  | |  | | 509/598 (85.1) | | |  | | |  | | | |  |
| Kandal | | *S*. *enterica* serovar Typhi | | | ≥2 y | | Outpatient | | ≥1 day of fever (≥38°C) | | 41/4985 (0.8) | | | Blood/Stool/ Culture | | | Kasper M. *et al.* 2010 | | | |  |
| Kampong Cham | | Dengue virus | | | 0-19 y | | Population survey (symptomatic) | | Fever | | Incidence rate per 1000 persons: | | | Blood/IgM ELISA (in-house), viral isolation, RT-PCR | | | Vong S. *et al.* 2010 | | | |  |
|  | | (2006) | | |  | |  | |  | | 13.4 | | |  | | |  | | | |  |
|  | | (2007) | | |  | |  | |  | | 57.8 | | |  | | |  | | | |  |
|  | | (2008) | | |  | |  | |  | | 17.6 | | |  | | |  | | | |  |
| Kampong Cham | | Dengue virus | | | <15 y | | In-and outpatient | | ≤7 days of fever or oral temp of ≥38°C | | 41/1000 (4.1) | | | Blood/IgM/IgG ELISA (in-house), viral isolation, RT-PCR | | | Wichmann O. *et al.* 2011 | | | |  |
| Siem Reap | | *S*. *enterica* serovar Typhi | | | <16 y | | Inpatient | | Fever of ≥38°C | | 5/134 (3.7) | | | Blood/IgM IFA, Culture, PCR | | | Wijedoru L. *et al.* 2011 | | | |  |
| **MYANMAR** | | | | | | | | | | | | | | | | | | | | |  |
| Yangon | | Dengue virus: | | | <10 y | | Inpatient | | Suspected dengue infection | |  | | | Blood/ Haemagglutination inhibition assay, plaque reduction neutralization assay, viral isolation | | Thein S. *et al.* 1997 | | | | |  |
|  | | (1984) | | |  | |  | |  | | 11/86 (12.8) | | |  | |  | | | | |  |
|  | | (1985) | | |  | |  | |  | | 8/65 (12.3) | | |  | |  | | | | |  |
|  | | (1986) | | |  | |  | |  | | 2/38 (5.3) | | |  | |  | | | | |  |
|  | | (1987) | | |  | |  | |  | | 6/99 (6.1) | | |  | |  | | | | |  |
|  | | (1988) | | |  | |  | |  | | 2/21 (9.5) | | |  | |  | | | | |  |
| Yangon | | Dengue virus | | | <1 y | | Inpatient | | Suspected dengue infection | | 449/8938 (5) | | | Blood/IgM/IgG ELISA (AFRIMS), haemagglutination inhibition assay | | Halstead S. *et al.* 2002 | | | | |  |
| Mandalay Region | | *S*. *enterica* serovar Typhi | | | All ages | | Not specified | | Suspected typhoid fever | | 3/49 (6.1) | | | Blood/Culture | | Aye T. *et al.* 2004 | | | | |  |
| **CHINA (YUNNAN PROVINCE)** | | | | | | | | | | | | | | | | | | | | |  |
| Yunnan | | *Orientia tsutsugamushi* | | | 18-66 y | | Population survey (symptomatic) | | Fever, headache, myalgia | | 8/13 (61.5) | | Blood/IFA, PCR | | | Zhang L. *et al.* 2007 | | | | |  |
|  | | *Rickettsia typhi* | | |  | |  | |  | | 5/13 (38.5) | | Blood/IFA, PCR | | |  | | | | |  |

^1^N/A: Information not available

^2^IFA: Indirect immunofluorescence assay; MAT: microscopic agglutination test; MCAT: micro capsule agglutination test; PCR: polymerase chain reaction; RT-PCR: reverse transcriptase-PCR; qPCR: real time PCR

^3^AST: aspartate transaminase

^4^ALT: alanine transaminase

^⧫^Asymptomatic population groups but with evidence of recent infection
