# Supplementary material for: Mapping the Aetiology of Non-Malarial Febrile Illness in Southeast Asia through a Systematic Review—Terra Incognita Impairing Treatment Policies
Source: PLoS One. 2012 Sep 6;7(9):e44269. doi: 10.1371/journal.pone.0044269 (PMC3435412; doi:10.1371/journal.pone.0044269)
Supplement: Table S2 — PubMed-Medline references for all papers of pathogen identification listed in Table S1. (DOCX) [file pone.0044269.s002.docx]

Table S2: PubMed-Medline references for all papers of pathogen identification listed in Table S1.

| Reference |
| --- |
| Anantapreecha S. et al 2005 http://www.ncbi.nlm.nih.gov/pubmed/15962557 |
| Anders K. et al 2011 http://www.ncbi.nlm.nih.gov/pubmed/21212214 |
| Appassakij H. et al 1995 http://www.ncbi.nlm.nih.gov/pubmed/7741173 |
| Aye T. et al 2004 http://www.ncbi.nlm.nih.gov/pubmed/15217177 |
| Bethell D. et al 1998 http://www.ncbi.nlm.nih.gov/pubmed/9498463 |
| Blacksell S. et al 2006 http://www.ncbi.nlm.nih.gov/pubmed/17028219 |
| Blacksell S. et al 2007 http://www.ncbi.nlm.nih.gov/pubmed/17715330 |
| Blacksell S. et al 2010 http://www.ncbi.nlm.nih.gov/pubmed/20682883 |
| Bounlu K. et al 1998 http://www.ncbi.nlm.nih.gov/pubmed/9798023 |
| Brown A. et al 1988 http://www.ncbi.nlm.nih.gov/pubmed/3124646 |
| Buchy P. et al 2005 http://www.ncbi.nlm.nih.gov/pubmed/15906664 |
| Chaowagul W. et al 1989 http://www.ncbi.nlm.nih.gov/pubmed/2708842 |
| Chau T. et al 2010 http://www.ncbi.nlm.nih.gov/pubmed/20405057 |
| Chhour Y. et al 2002 http://www.ncbi.nlm.nih.gov/pubmed/11996683 |
| Chinh N. et al 2000 http://www.ncbi.nlm.nih.gov/pubmed/10858343 |
| Chokephaibulkit K. et al 2001 http://www.ncbi.nlm.nih.gov/pubmed/11224846 |
| Cohen A. et al 2007 http://www.ncbi.nlm.nih.gov/pubmed/17207147 |
| Dolecek C. et al 2008 http://www.ncbi.nlm.nih.gov/pubmed/18493312 |
| Duffy P. et al 1990 http://www.ncbi.nlm.nih.gov/pubmed/2122749 |
| Duyen HT. et al 2011 http://www.ncbi.nlm.nih.gov/pubmed/21335562 |
| Eamsila C. et al 1996 http://www.ncbi.nlm.nih.gov/pubmed/8940989 |
| Ekpo P. et al 2007 http://www.ncbi.nlm.nih.gov/pubmed/17428952 |
| Ellis R. et al 2006 http://www.ncbi.nlm.nih.gov/pubmed/16407353 |
| Endy T. et al 2002 http://www.ncbi.nlm.nih.gov/pubmed/12076887 |
| Fox A. et al 2011 http://www.ncbi.nlm.nih.gov/pubmed/21390156 |
| Frances S. et al 1997 http://www.ncbi.nlm.nih.gov/pubmed/9561627 |
| Halstead S. et al 2002 http://www.ncbi.nlm.nih.gov/pubmed/12498666 |
| Halstead S. et al 2002 http://www.ncbi.nlm.nih.gov/pubmed/12498666 |
| Halstead S. et al 2002 http://www.ncbi.nlm.nih.gov/pubmed/12498666 |
| Hang V. et al 2009 http://www.ncbi.nlm.nih.gov/pubmed/19156192 |
| Hoa N. et al 1998 http://www.ncbi.nlm.nih.gov/pubmed/9861362 |
| Hongsiriwon S. et al 2002 http://www.ncbi.nlm.nih.gov/pubmed/12118460 |
| Huis In't Veld D. et al 2005 http://www.ncbi.nlm.nih.gov/pubmed/16222004 |
| Huy R. et al 2010 http://www.ncbi.nlm.nih.gov/pubmed/20865069 |
| KaN/Aphun P. et al 1993 http://www.ncbi.nlm.nih.gov/pubmed/7678106 |
| Kasper M. et al 2010 http://www.ncbi.nlm.nih.gov/pubmed/19800753 |
| Kemapunmanus M. et al 2004 http://www.ncbi.nlm.nih.gov/pubmed/15916082 |
| Kittigul L. et al 2002 http://www.ncbi.nlm.nih.gov/pubmed/11957027 |
| Kittigul L. et al 2003 http://www.ncbi.nlm.nih.gov/pubmed/12971568 |
| Kowitdamrong E. et al 2001 http://www.ncbi.nlm.nih.gov/pubmed/11529328 |
| Kramme S. et al 2009 http://www.ncbi.nlm.nih.gov/pubmed/19144812 |
| Lapphra K. et al 2008 http://www.ncbi.nlm.nih.gov/pubmed/18191361 |
| Laras K. et al 2002 http://www.ncbi.nlm.nih.gov/pubmed/12408667 |
| Laras K. et al 2002 http://www.ncbi.nlm.nih.gov/pubmed/12408667 |
| Laras K. et al 2002 http://www.ncbi.nlm.nih.gov/pubmed/12408667 |
| Le V. et al 2010 http://www.ncbi.nlm.nih.gov/pubmed/21049060 |
| Libraty D. et al 2007 http://www.ncbi.nlm.nih.gov/pubmed/18160980 |
| Limmathurotsakul D. et al 2005 http://www.ncbi.nlm.nih.gov/pubmed/15872255 |
| Limmathurotsakul D. et al 2010 http://www.ncbi.nlm.nih.gov/pubmed/20519609 |
| Lin F. et al 2000 http://www.ncbi.nlm.nih.gov/pubmed/11289678 |
| Mayxay M. et al 2011 http://www.ncbi.nlm.nih.gov/pubmed/20958892 |
| McGready R. et al 2010 http://www.ncbi.nlm.nih.gov/pubmed/21103369 |
| Myint K. et al 2007 http://www.ncbi.nlm.nih.gov/pubmed/17255242 |
| Nguyen N. et al 1997 http://www.ncbi.nlm.nih.gov/pubmed/9579614 |
| Nguyen T. et al 2005 http://www.ncbi.nlm.nih.gov/pubmed/15827272 |
| Nisalak A. et al 2003 http://www.ncbi.nlm.nih.gov/pubmed/12641411 |
| Niwattayakul K. et al 2002 http://www.ncbi.nlm.nih.gov/pubmed/12118444 |
| Niwetpathomwat A. et al 2005 http://www.ncbi.nlm.nih.gov/pubmed/16438210 |
| Olsen S. et al 2010 http://www.ncbi.nlm.nih.gov/pubmed/20674433 |
| Pancharoen C. et al 2001 http://www.ncbi.nlm.nih.gov/pubmed/11944702 |
| Parola P. et al 2003 http://www.ncbi.nlm.nih.gov/pubmed/12737744 |
| Parry C. et al 1999 http://www.ncbi.nlm.nih.gov/pubmed/10449469 |
| Parry C. et al 1999 http://www.ncbi.nlm.nih.gov/pubmed/10524986 |
| Phetsouvanh R. et al 2006 http://www.ncbi.nlm.nih.gov/pubmed/17124000 |
| Phetsouvanh R. et al 2009 http://www.ncbi.nlm.nih.gov/pubmed/19407134 |
| Phongmany S. et al 2006 http://www.ncbi.nlm.nih.gov/pubmed/16494751 |
| Phraisuwan P. et al 2002 http://www.ncbi.nlm.nih.gov/pubmed/12498663 |
| Phuong C. et al 2004 http://www.ncbi.nlm.nih.gov/pubmed/14993629 |
| Phuong H. et al 2006 http://www.ncbi.nlm.nih.gov/pubmed/16869969 |
| Phuong H. et al 2010 http://www.ncbi.nlm.nih.gov/pubmed/20858230 |
| Poblap T. et al 2006 http://www.ncbi.nlm.nih.gov/pubmed/17333731 |
| PradutkanchaN/A J. et al 2003 http://www.ncbi.nlm.nih.gov/pubmed/12971532 |
| Rathavuth H. et al 1997 http://www.ncbi.nlm.nih.gov/pubmed/9322294 |
| Reechaipichitkul W. 2004 http://www.ncbi.nlm.nih.gov/pubmed/15689084 |
| Seng H et al 2007 http://www.ncbi.nlm.nih.gov/pubmed/17427534 |
| Silpapojakul K. 1991 http://www.ncbi.nlm.nih.gov/pubmed/2041666 |
| Singhsilarak T. et al 2006 http://www.ncbi.nlm.nih.gov/pubmed/16771204 |
| SirisanthaN/A T. et al 1994 http://www.ncbi.nlm.nih.gov/pubmed/8024059 |
| Smythe L. et al 2009 http://www.ncbi.nlm.nih.gov/pubmed/19815889 |
| Solomon T. et al 1998 http://www.ncbi.nlm.nih.gov/pubmed/9650956 |
| Solomon T. et al 2002 http://www.ncbi.nlm.nih.gov/pubmed/11960897 |
| Solomon T. et al 2008 http://www.ncbi.nlm.nih.gov/pubmed/18368204 |
| Sonthayanon P. et al 2006 http://www.ncbi.nlm.nih.gov/pubmed/17172374 |
| Srey V. et al 2002 http://www.ncbi.nlm.nih.gov/pubmed/12135294 |
| Srikiatkhachorn A. et al 2010 http://www.ncbi.nlm.nih.gov/pubmed/20205587 |
| Sripanidkulchai R. et al 2005 http://www.ncbi.nlm.nih.gov/pubmed/16438152 |
| Strickman D. et al 1994 http://www.ncbi.nlm.nih.gov/pubmed/8074248 |
| Strickman D. et al 2000 http://www.ncbi.nlm.nih.gov/pubmed/11357991 |
| Suputtamongkol Y. et al 1994 http://www.ncbi.nlm.nih.gov/pubmed/7860160 |
| Suputtamongkol Y. et al 2004 http://www.ncbi.nlm.nih.gov/pubmed/15546074 |
| Suputtamongkol Y. et al 2009 http://www.ncbi.nlm.nih.gov/pubmed/19538278 |
| Suttinont C. et al 2006 http://www.ncbi.nlm.nih.gov/pubmed/16762116 |
| Syhavong B. et al 2010 http://www.ncbi.nlm.nih.gov/pubmed/20378138 |
| TangkaN/Akul W. et al 2000 http://www.ncbi.nlm.nih.gov/pubmed/11388516 |
| Tansuphasiri U. et al 2004 http://www.ncbi.nlm.nih.gov/pubmed/15916034 |
| TantitaN/Awat S. et al 2003 http://www.ncbi.nlm.nih.gov/pubmed/14650704 |
| Thai K. et al 2007 http://www.ncbi.nlm.nih.gov/pubmed/18076564 |
| Thai K. et al 2010 http://www.ncbi.nlm.nih.gov/pubmed/20080126 |
| Thaipadungpanit J. et al 2007 http://www.ncbi.nlm.nih.gov/pubmed/17989782 |
| Thein S. et al 1997 http://www.ncbi.nlm.nih.gov/pubmed/9180609 |
| Tien N. et al 2010 http://www.ncbi.nlm.nih.gov/pubmed/20630553 |
| Touch S. et al 2009 http://www.ncbi.nlm.nih.gov/pubmed/19747185 |
| Tran H. et al 2005 http://www.ncbi.nlm.nih.gov/pubmed/16099488 |
| Tran T. et al 1995 http://www.ncbi.nlm.nih.gov/pubmed/7795095 |
| Tran T. et al 2006 http://www.ncbi.nlm.nih.gov/pubmed/16436203 |
| Trung D. et al 2010 http://www.ncbi.nlm.nih.gov/pubmed/20889864 |
| Vallée J. et al 2009 http://www.ncbi.nlm.nih.gov/pubmed/19563430 |
| Van Benthem B. et al 2005 http://www.ncbi.nlm.nih.gov/pubmed/15741558 |
| Van C. et al 1998 http://www.ncbi.nlm.nih.gov/pubmed/10326104 |
| Vaughn D. et al 1997 http://www.ncbi.nlm.nih.gov/pubmed/9237696 |
| Vaughn D. et al 2000 http://www.ncbi.nlm.nih.gov/pubmed/10608744 |
| Vinh H. et al 1996 http://www.ncbi.nlm.nih.gov/pubmed/8849259 |
| Vong S. et al 2010 http://www.ncbi.nlm.nih.gov/pubmed/21152061 |
| Wain J. et al 2001 http://www.ncbi.nlm.nih.gov/pubmed/11283089 |
| Walsh A. et al 1995 http://www.ncbi.nlm.nih.gov/pubmed/8749644 |
| Watt G. et al 1998 http://www.ncbi.nlm.nih.gov/pubmed/9498469 |
| Watt G. et al 2003 http://www.ncbi.nlm.nih.gov/pubmed/12887030 |
| Wichmann O. et al 2004 http://www.ncbi.nlm.nih.gov/pubmed/15361117 |
| Wichmann O. et al 2011 http://www.ncbi.nlm.nih.gov/pubmed/21468308 |
| Wichmann O. *et al*. 2011 http://www.ncbi.nlm.nih.gov/pubmed/21468308 |
| Wijedoru L. et al 2011 http://www.ncbi.nlm.nih.gov/pubmed/21508082 |
| Wilde H. et al 1991 http://www.ncbi.nlm.nih.gov/pubmed/1812601 |
| Wuthiekanun V. et al 2005 http://www.ncbi.nlm.nih.gov/pubmed/15817767 |
| Wuthiekanun V. et al 2007 http://www.ncbi.nlm.nih.gov/pubmed/17301285 |
| Wuthiekanun V. et al 2007 http://www.ncbi.nlm.nih.gov/pubmed/17370525 |
| Yoksan S. et al 2009 http://www.ncbi.nlm.nih.gov/pubmed/19800560 |
| Zhang L. et al 2007 http://www.ncbi.nlm.nih.gov/pubmed/17711736 |
