# Supplementary material for: Mapping the Aetiology of Non-Malarial Febrile Illness in Southeast Asia through a Systematic Review—Terra Incognita Impairing Treatment Policies
Source: PLoS One. 2012 Sep 6;7(9):e44269. doi: 10.1371/journal.pone.0044269 (PMC3435412; doi:10.1371/journal.pone.0044269)
Supplement: Table S3 — Frequency of past infection with pathogen known to cause fever in the Mekong region: location, sample type, and laboratory tests of studies from 1991 to 2010. (DOCX) [file pone.0044269.s003.docx]

Table S3: Frequency of past infection with pathogen known to cause fever in the Mekong region: location, sample type, and laboratory tests of studies from 1991 to 2010 are shown.

| **Location(s)** | **Pathogens (date)** | **Positive/Tested samples (%)** | **Test(s) used** | **Sample(s)** | **Reference** |
| --- | --- | --- | --- | --- | --- |
| **THAILAND** | | | | | |
| Chiang Rai | *Rickettsia japonica* | 11/122 (9.0) | Indirect Immunoperoxidase IgG/IgM assay | Blood | Takada et al, 1993 ; http://www.ncbi.nlm.nih.gov/pubmed/8266242 |
|  | *Rickettsia* ssp | 13/122 (10.7) |  |  |  |
|  | *Rickettsia typhi* | 3/122 (2.5) |  |  |  |
|  | Thai TT-118 | 26/122 (21.3) |  |  |  |
|  | *Orientia tsutsugamushi* |  | IFA | Blood | Eamsila et al, 1996 ; http://www.ncbi.nlm.nih.gov/pubmed/8940989 |
| Utraladit | (April 1989) | 11/80 (13.8) |  |  |  |
|  | (July 1989) | 4/51 (7.8) |  |  |  |
| Phitsanulok | (April 1989) | 58/681 (8.5) |  |  |  |
|  | (July 1989) | 23/380 (6.1) |  |  |  |
| Ubon Ratchathani | (April 1989) | 25/341 (7.3) |  |  |  |
|  | (July 1989) | 21/230 (9.1) |  |  |  |
| Buriram | (April 1989) | 15/171 (8.8) |  |  |  |
|  | (July 1989) | 9/164 (5.5) |  |  |  |
| Prachinburi | (April 1989) | 35/299 (11.7) |  |  |  |
|  | (July 1989) | 16/165 (9.7) |  |  |  |
| Tak | (April 1989) | 25/211 (11.8) |  |  |  |
|  | (July 1989) | 7/153 (4.6) |  |  |  |
| Kanchanaburi | (April 1989) | 26/105 (24.8) |  |  |  |
|  | (July 1989) | 15/75 (20.0) |  |  |  |
| Ranong | (July 1991) | 4/78 (5.1) |  |  |  |
| Surathani | (July 1991) | 10/147 (6.8) |  |  |  |
| Songkhla | (July 1991) | 1/62 (1.6) |  |  |  |
| Yala | (July 1991) | 7/182 (3.8) |  |  |  |
| Narathiwat | July 1991 | 3/100 (3.0) |  |  |  |
| Sisaket | *Orientia tsutsugamushi* |  | IFA | Blood | Frances et al, 1997 ; http://www.ncbi.nlm.nih.gov/pubmed/9561627 |
|  | (September 1991) | 6/333 (1.8) |  |  |  |
|  | (January 1992) | 9/270 (3.3) |  |  |  |
|  | (April 1992) | 0/292 (0) |  |  |  |
|  | (September 1992) | 10/244 (4.1) |  |  |  |
| Ratchaburi; Kanchanaburi; Phetchaburi | *Orientia tsutsugamushi* | 119/200 (59.5) | IFA | Blood | Chanyasanha et al, 1998 ; http://www.ncbi.nlm.nih.gov/pubmed/9876950 |
| Ratchaburi | Dengue virus | 200/283 (70.7) | IgM/IgG ELISA (in-house) | Blood | Tuntaprasart et al, 2003 ; http://www.ncbi.nlm.nih.gov/pubmed/15115129 |
| Nakhon Ratchasima | *Aeromonas hydrophila* | 1/36 (2.8) | Culture | Blood | Leelarasamme et al, 2004 ; http://www.ncbi.nlm.nih.gov/pubmed/15222513 |
|  | *Burkholderia pseudomallei* | 11/1137 (1.0) | IFA |  |  |
|  | Chikungunya virus | 14/1137 (1.2) | IgM ELISA/HIA |  |  |
|  | Dengue virus | 70/1137 (6.2) | IgM ELISA (in-house)/HIA |  |  |
|  | *Enterobacter* spp | 4/36 (11.1) | Culture |  |  |
|  | *Escherichia coli* | 13/36 (36.1) | Culture |  |  |
|  | Influenza | 73/1137 (6.4) | HIA |  |  |
|  | Japanese encephalitis | 7/1137 (0.6) | IgM ELISA/HIA |  |  |
|  | *Klebsiella pneumoniae* | 1/36 (2.8) | Culture |  |  |
|  | *Leptospira* | 14/1137 (1.2) | MAT |  |  |
|  | *Orientia tsutsugamushi* | 91/1137 (8.0) | Weil-Felix test |  |  |
|  | *Rickettsia typhi* | 65/1137 (5.7) | Weil.Felix test |  |  |
|  | *S*. *enterica* serovar Typhi | 3/36 (8.3) | Culture |  |  |
|  | *Salmonella* ssp | 23/1137 (2.0) | Widal test |  |  |
|  | *Staphylococcus aureuas* | 4/36 (11.1) | Culture |  |  |
|  | *Streptococcus* spp | 6/36 (16.7) | Culture |  |  |
| Bangkok | Dengue virus | 49/93 (52.7) | IgM/IgG ELISA (AFRIMS) | Blood | Mekmullica et al, 2005 ; http://www.ncbi.nlm.nih.gov/pubmed/15906667 |
| Phang Nga | *Burkholderia pseudomallei* | 181/2039 (8.9) | IHA | Blood | Wuthiekanun et al, 2006 ; http://www.ncbi.nlm.nih.gov/pubmed/16390980 |
| Chachoengsao | Dengue virus |  | IgM/IgG ELISA (Innis) | Blood | Kittayapong et al, 2008 ; http://www.ncbi.nlm.nih.gov/pubmed/18187787 |
|  | Year 1 | 30/1625 (1.9) |  |  |  |
|  | Year 2 | 118/1755 (6.7) |  |  |  |
| North | *Burkholderia pseudomallei* | 566/4019 (14.1) | Culture | Blood/Pus | Paveenkittiporn et al, 2009 ; http://www.ncbi.nlm.nih.gov/pubmed/21298845 |
| Northeast |  | 2776/4019 (69.1) |  |  |  |
| Center |  | 475/4019 (11.8) |  |  |  |
| South |  | 202/4019 (5.0) |  |  |  |
| **VIET NAM** |  |  |  |  |  |
| Duyen Thai (Hanoi) | *S*. *enterica* serovar Typhi | 97/138 (70.3) | Widal test | Blood | Prokopec et al, 1991; http://www.ncbi.nlm.nih.gov/pubmed/1880411 |
| Mai Chau (Hòa Bìn) | *S*. *enterica* serovar Typhi | 72/154 (46.8) |  |  |  |
| Dong Thap | Flavivirus | 203/308 (65.9) | IgG ELISA | Blood | Bartley et al, 2002 ; http://www.ncbi.nlm.nih.gov/pubmed/12002539 |
| Binh Thuan | Dengue virus | 631/961 (65.7) | IgM/IgG ELISA (Focus) | Blood | Thai et al, 2005 ; http://www.ncbi.nlm.nih.gov/pubmed/15807802 |
| Binh Thuan | *Leptospira* | 123/961 (12.8) | IgG/IgM ELISA | Blood | Thai et al, 2006 ; http://www.ncbi.nlm.nih.gov/pubmed/16640627 |
| Ho Chi Minh | Dengue virus | 10/819 (1.2) | IgM/IgG ELISA Plaque reduction neutralization assay | Blood | Chau et al, 2009 ; http://www.ncbi.nlm.nih.gov/pubmed/19911991 |
| **LAO PDR** |  |  |  |  |  |
| Vientiane; Luang Prabang; Bolikhamsai; Champassack | *Leptospira* | 763/2083 (36.6) | IgG ELISA | Blood | Laras et al, 2002 ; http://www.ncbi.nlm.nih.gov/pubmed/12408667 |
| Attapeu | Dengue virus | 1/225 (0.4) | IgM/IgG ELISA (PanBio) | Blood | Peyerl-Hoffmann et al, 2004 ; http://www.ncbi.nlm.nih.gov/pubmed/14759246 |
|  | *Plasmodium falciparum* | 26/225 (11.6) | Microscopy, Dipstick test |  |  |
| Khammouane | *Leptospira* | 96/406 (23.7) | MAT | Blood | Kawaguchi et al, 2008 ; http://www.ncbi.nlm.nih.gov/pubmed/18541776 |
| Vientiane; Savannakhet | *Orientia tsutsugamushi* | 170/1132 (15.0) | IFA, Western blot | Blood | Newton et al, 2009 ; http://www.ncbi.nlm.nih.gov/pubmed/19635868 |
| Vientiane | Japanese Encephalitis virus: |  | IgG ELISA | Blood | Vallée et al, 2009http://www.ncbi.nlm.nih.gov/pubmed/19563430 |
|  | Adults | 1686/1990(84.6) |  |  |  |
|  | Children | 147/1568 (9.4) |  |  |  |
| Vientiane | *Orientia tsutsugamushi* | 394/2002 (19.7) | IgG ELISA (PanBio) | Blood | Vallée et al, 2010, http://www.ncbi.nlm.nih.gov/pubmed/21151880 |
|  | *Rickettsia typhi* | 440/2002 (22.0) |  |  |  |
| **CAMBODIA** |  |  |  |  |  |
| Siem Reap | *Burkholderia pseudomallei* | 159/968 (16.4) | IHA | Blood | Wuthiekanun et al, 2008 ; http://www.ncbi.nlm.nih.gov/pubmed/18258125 |
| **MYANMAR** |  |  |  |  |  |
| Kayin State; Mon State; Bago Division; Yangon Region | *Burkholderia pseudomallei* | 757/968 (78.2) | IHA | Blood | Wuthiekanun et al, 2006 ; http://www.ncbi.nlm.nih.gov/pubmed/17123993 |
